# Developing Graphene‐Based Moiré Heterostructures for Twistronics

**DOI:** 10.1002/advs.202103170

**Published:** 2021-11-01

**Authors:** Mengya Liu, Liping Wang, Gui Yu

**Affiliations:** ^1^ School of Materials Science and Engineering University of Science and Technology Beijing Beijing 100083 P. R. China; ^2^ Beijing National Laboratory for Molecular Sciences CAS Research/Education Center for Excellence in Molecular Sciences Institute of Chemistry Chinese Academy of Sciences Beijing 100190 P. R. China; ^3^ School of Chemical Sciences University of Chinese Academy of Sciences Beijing 100049 P. R. China

**Keywords:** correlated physical performances, graphene‐based moiré heterostructures, topological properties, twist angles, twistronics

## Abstract

Graphene‐based moiré heterostructures are strongly correlated materials, and they are considered to be an effective platform to investigate the challenges of condensed matter physics. This is due to the distinct electronic properties that are unique to moiré superlattices and peculiar band structures. The increasing research on strongly correlated physics via graphene‐based moiré heterostructures, especially unconventional superconductors, greatly promotes the development of condensed matter physics. Herein, the preparation methods of graphene‐based moiré heterostructures on both in situ growth and assembling monolayer 2D materials are discussed. Methods to improve the quality of graphene and optimize the transfer process are presented to mitigate the limitations of low‐quality graphene and damage caused by the transfer process during the fabrication of graphene‐based moiré heterostructures. Then, the topological properties in various graphene‐based moiré heterostructures are reviewed. Furthermore, recent advances regarding the factors that influence physical performances via a changing twist angle, the exertion of strain, and regulation of the dielectric environment are presented. Moreover, various unique physical properties in graphene‐based moiré heterostructures are demonstrated. Finally, the challenges faced during the preparation and characterization of graphene‐based moiré heterostructures are discussed. An outlook for the further development of moiré heterostructures is also presented.

## Graphical abstract

This review presents the preparation and optimization of graphene‐based moiré heterostructures, including twisted bilayer graphene, twisted trilayer graphene, twisted double bilayer graphene, graphene/ hexagonal boron nitride, and graphene/transition metal dichalcogenides. The topological properties and correlated physical performances are comprehensively illustrated. A clear understanding of the mechanism regarding many‐body physics will be a breakthrough for condensed matter physics.

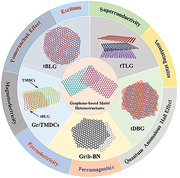

## 1 Introduction

The discovery of graphene with superior physical, perfect optical, and unique electronic properties has triggered extensive interest in the field of condensed matter physics.^[^ ^1^ ^]^ As the simplest of the graphene materials, monolayer graphene (MLG) has a singular energy band structure and exhibits outstanding electronic performance.^[^ ^2^ ^]^ Apart from graphene, 2D materials, such as hexagonal boron nitride (*h*‐BN)^[^ ^3^ ^]^ and transition metal dichalcogenides (TMDCs),^[^ ^4^ ^]^ have been extensively researched, owing to their excellent electronic properties or intrinsic magnetic order. Additionally, these 2D materials may be used to fabricate vertical van der Waals (vdW) heterostructures, owing to the weak vdW interactions between layers. The unique and engineered structures of vertical vdW heterostructures with diverse physical properties benefited research on condensed matter physics.^[^ ^5^, ^6^, ^7^ ^]^ Now, graphene‐based moiré heterostructures, which are formed by stacking bilayer graphene (BLG), multilayer graphene, or graphene with other 2D materials, are the representative vertical vdW heterostructures.^[^ ^8^, ^9^, ^10^ ^]^ Graphene‐based moiré heterostructures exhibited topological properties caused by a moiré superlattice and various correlated states, which are introduced by strong electron–electron interactions.^[^ ^11^, ^12^ ^]^ Since the discovery of unconventional superconductivity, correlated insulator behavior in magic‐angle bilayer graphene (MATBG) and the transition from insulation to superconductivity were simply realized. Thus, graphene‐based moiré heterostructures were introduced to the field of strong correlation physics, thereby enabling research on unconventional superconductivity.^[^ ^10^, ^13^ ^]^ Inspired by these discoveries, there is an upsurge in the study of twisted trilayer graphene (tTLG),^[^ ^14^, ^15^ ^]^ twisted double bilayer graphene (tDBG),^[^ ^16^ ^]^ twisted graphene/*h*‐BN,^[^ ^5^ ^]^ and twisted graphene/TMDCs^[^ ^17^ ^]^ heterostructures. That is, the studies on graphene‐based moiré heterostructures triggered significant passion of researchers.

Currently, the preparation methods of graphene‐based moiré heterostructures include in situ growth^[^ ^18^, ^19^ ^]^ and self‐assembly via stacking or folding monolayer 2D materials.^[^ ^20^ ^]^ However, neither method can be perfectly implemented. The in situ growth method represented by chemical vapor deposition (CVD) cannot produce graphene‐based moiré heterostructures with a precisely controlled twist angle. The self‐assembling characteristic of 2D materials enables a more precise twist angle; however, it is easy to introduce contamination, wrinkles, and polymer residues during the transfer process. One method to fabricate high‐quality graphene‐based moiré heterostructures is to develop an effective CVD growth method by regulating the growth conditions; another way is to optimize the assembling process. Extensive research was conducted on assembling because it is currently the most feasible method; this included optimizing the transfer process^[^ ^21^ ^]^ to reduce unnecessary damage and improving the quality of graphene^[^ ^22^ ^]^ to decrease surface contamination. Clearly, it is difficult to precisely control the twisted moiré structures, which hinders the rapid progress of graphene‐based moiré heterostructures. Therefore, we summarize existing preparation methods to provide a guide for the development of graphene‐based moiré heterostructures.

In addition to the preparation of graphene‐based moiré heterostructures, many recent studies focused on the observation of their topological and correlated properties, such as, the existence of a moiré superlattice and topological minibands,^[^ ^10^, ^17^, ^23^, ^24^, ^25^ ^]^ the quantum anomalous Hall effect in twisted bilayer graphene (tBLG),^[^ ^26^ ^]^ the Mott insulator in ABC‐TLG,^[^ ^24^ ^]^ the ferromagnetism in tDBG,^[^ ^16^ ^]^ the Pomeranchuk effect in MATBG,^[^ ^27^ ^]^ the ferroelectricity in BLG/*h*‐BN,^[^ ^28^ ^]^ and the exciton effect in graphene/TMDCs heterostructures.^[^ ^29^ ^]^ A few reviews on the physical properties and applications have been reported; however, they are primarily focused on tBLG and TMDCs vdW heterostructures.^[^ ^30^, ^31^, ^32^, ^33^ ^]^ Currently, there was no review on the systematic description of moiré patterns appearing in heterostructures. Moreover, various newly discovered physical properties in graphene‐based moiré heterostructures have not been systematically summarized. The preparation, quality improvement and topological properties of graphene‐based moiré heterostructures and the factors that influence their physical properties, such as twist angle, strain, and dielectric environment, lack detailed descriptions in the literature. Herein, we first present the preparation methods used for graphene‐based moiré heterostructures; this includes in situ growth and assembling using monolayer 2D materials. Subsequently, we address the limitations of the preparation methods by discussing and summarizing how to optimize the transfer process and improve the quality of graphene. Further, topological properties are discussed to present various moiré patterns and corresponding energy band structures. Moreover, the factors that influence the physical properties of graphene‐based moiré heterostructures are presented, and they more precisely tune the performance. Meanwhile, the typical performance of superconductivity, the correlated insulator and various newly observed properties are systematically introduced; these include the quantum anomalous Hall effect,^[^ ^26^ ^]^ ferromagnetism,^[^ ^34^ ^]^ ferroelectricity,^[^ ^28^ ^]^ the Pomeranchuk effect,^[^ ^27^ ^]^ and tunable excitons.^[^ ^35^ ^]^ Finally, we summarize the development of twistronics based on graphene‐based moiré heterostructures, including the development limitations in fabrication, characterization, and a deep investigation into the mechanism of many‐body physics.

## 2 Preparation of Graphene‐Based Moiré Heterostructures

Graphene‐based moiré heterostructures consist of BLG, multilayer graphene or graphene flakes, and other 2D materials with weak vdW forces between the layers, such as tBLG,^[^ ^36^ ^]^ TLG,^[^ ^37^ ^]^ tDBG,^[^ ^38^ ^]^ and graphene/X^[^ ^39^, ^40^ ^]^ (X = *h*‐BN, TMDCs). In the field of artificial graphene‐based moiré heterostructures, the most important process is the effective preparation of moiré superlattices with specific alignment. According to the processes, the preparation methods can be divided into two categories: in situ growth and assembling of monolayer 2D material. In this section, a detailed introduction on the preparation method of graphene‐based moiré heterostructures is presented.

### 2.1 In Situ Growth

**Figure 1:**
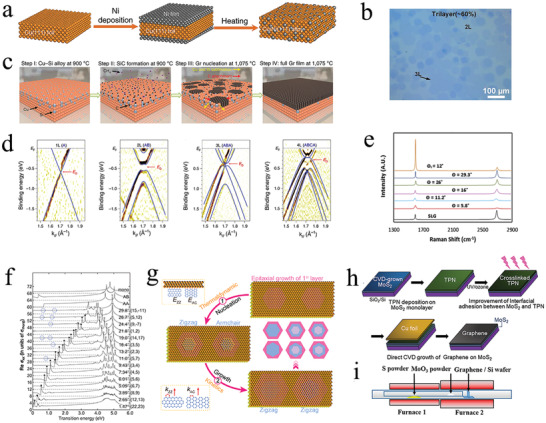
The in situ growth of graphene‐based moiré heterostructures. a) Schematic illustration of the preparation of Cu/Ni alloy. b) Optical image of graphene grown on Cu/Ni alloy with 20.3 at% Ni concentrations. a,b) Reproduced with permission.^[^ ^44^ ^]^ Copyright 2020, Springer Nature. c) Schematic images of the graphene growth steps in Cu/Si alloy. d) Nano‐ARPES spectra of monolayer, bilayer, trilayer, and tetralayer graphene. The blue lines represent corresponding DFT calculations. c,d) Reproduced with permission.^[^ ^45^ ^]^ Copyright 2020, Springer Nature. e) Raman spectra of MLG and tBLG with different twist angles. Reproduced with permission.^[^ ^50^ ^]^ Copyright 2017, Elsevier Ltd. f) Dynamical conductivities of tBLG with various twist angles. Reproduced with permission.^[^ ^53^ ^]^ Copyright 2013, American Chemical Society. g) Schematic illustration of the tBLG growth on Cu (111) via a designed two‐step epitaxial growth process. Reproduced with permission.^[^ ^18^ ^]^ Copyright 2020, American Chemical Society. h) Schematic illustration of the preparation process of graphene/MoS_2_ heterostructure on SiO_2_/Si substrate. Reproduced with permission.^[^ ^54^ ^]^ Copyright 2020, American Chemical Society. i) Schematic illustration of the CVD system used to synthesize graphene/MoS_2_ heterostructure. Reproduced with permission.^[^ ^55^ ^]^ Copyright 2018, American Chemical Society.

The stacking order between different layers of graphene‐based moiré heterostructures has a crucial influence on their physical properties. For example, when the twist angle is approximately the magic angle, the band structures in BLG becomes flat, thereby resulting in unconventional superconductivity and insulating states.^[^ ^12^, ^13^ ^]^ Thus, the key to the research of novel physical properties in graphene‐based moiré heterostructures is precise preparation. The in situ growth method, without the aid of a transfer process, enables high quality products and avoids the introduction of impurities and wrinkles. In situ growth includes CVD and epitaxial growth. Recently, there were many competing studies on the preparation of graphene‐based moiré heterostructures via CVD, owing to the many advantages of this method, such as the high cleanliness and feasible transfer, which benefits research on intrinsic performance in more depth.^[^ ^41^, ^42^ ^]^ As an example, it is difficult to grow TLG on Cu foils using the CVD method, owing to the low amount of dissolved carbon and self‐limiting growth.^[^ ^43^ ^]^ Moreover, the carbon solubility on Ni is larger than that on Cu, and the carbon may be completely dissolved in the Cu/Ni alloy under suitable conditions (**Figure** **1**a).^[^ ^44^ ^]^ Based on this, the preparation of ABA‐stacked graphene was experimentally investigated on a Cu/Ni alloy by changing the composition of Cu and Ni to achieve finite carbon solubility.^[^ ^44^ ^]^ In the Cu/Ni alloy, the content of Ni directly determined the carbon solubility. As a result, the number of graphene layers increased with the Ni content in the alloy. ABA‐TLG coverage of ≈60% was obtained when the Ni content increased to 20.3 at% (Figure 1b). Similarly, the CVD method was proposed for Cu/Si alloy to control the layer thickness and stacking order by regulating the Ni content.^[^ ^45^ ^]^ From the illustration of the growth steps (Figure 1c), it can be observed that the Cu/Si alloy was formed under a H_2_‐rich environment, the quartz chamber was etched, Si atoms were introduced, and SiC formed when CH_4_ was brought into the system. The carbon atoms precipitated to form graphene as the Si atoms sublimated with increasing temperature. The characterization via angle‐resolved photoemission spectroscopy (nano‐ARPES) showed perfect consistency between experimental and theoretical calculations (Figure 1d). Thus, uniformly oriented bilayer (AB), trilayer (ABA), and tetralayer (ABCA) graphene were successfully prepared. Furthermore, Raman spectroscopy is a nondestructive characterization tool, and it is also a good method to characterize the orientation^[^ ^46^, ^47^, ^48^ ^]^ and number of layers^[^ ^49^ ^]^ of graphene‐based moiré heterostructures. Considering tBLG as an example, the peak ratio of 2D/G decreases as the number of layers increases; the full width at half maximum of the 2D peak is positively related to the twist angle under the condition of a twist angle less than 12°, and the situation is reversed for a twist angle larger than 12° (Figure 1e).^[^ ^50^ ^]^ Additionally, the trends observed in the spectroscopic characteristics of tBLG may serve as a fingerprint to identify the twist angle.^[^ ^51^, ^52^ ^]^ The peak structure continuously changes with the twist angle (Figure 1f).^[^ ^53^ ^]^ Based on optical spectroscopy, we can approximate the orientation of graphene‐based moiré heterostructures. However, the primary aim is still the preparation of high‐quality graphene‐based moiré heterostructures.

Although the above‐mentioned CVD‐grown graphene‐based moiré heterostructures have many advantages, it is difficult to accurately tune the interlayer twist angle between different 2D materials. The fundamental reason is that there is interlayer interaction during the CVD growth process. To decrease the interlayer coupling, tBLG was fabricated on Cu (111) via a designed two‐step epitaxial growth process (Figure 1g).^[^ ^18^ ^]^ The orientation was limited to 0° and 30° because there was a good epitaxial relationship between Cu (111) and graphene.^[^ ^56^ ^]^ In the stage of nucleation, the graphene/Cu step interface determined the graphene orientation.^[^ ^57^ ^]^ Moreover, the second layer started to nucleate with the carbon atoms diffused underneath the graphene edge. In this method, the number of layers and morphology were controlled by the thermodynamically dominated nucleation and kinetically regulated growth, which enabled the in‐situ preparation of graphene‐based moiré heterostructures.

In addition to the preparation of twisted graphene, the CVD process can also realize the preparation of graphene/X (X = *h*‐BN, TMDCs) heterostructures, which provides a platform to explore the novel electronic interactions and potential applications in thermoelectric nanodevices,^[^ ^40^ ^]^ spin information transport,^[^ ^58^ ^]^ and integrated photodetectors.^[^ ^59^ ^]^ The CVD preparation methods for the two types of graphene/X heterostructures are similar. That is, graphene was first grown on a substrate, and then *h*‐BN or TMDCs was prepared, or vice versa. Taking the graphene/TMDCs heterostructures as examples, we describe the preparation of graphene/X heterostructures below.

Briefly, the direct CVD preparation of graphene/MoS_2_ heterostructures is as follows (Figure 1h).^[^ ^54^ ^]^ First, MoS_2_ was prepared on a Si/SiO_2_ substrate via CVD. Second, a solid carbon source [1,2,3,4‐tetraphenylnaphthalene (TPN)] was spin‐coated on MoS_2_ and then exposed to ultraviolet/ozone to increase its interfacial adhesion. Finally, Cu was placed on TPN to grow graphene via CVD. Consequently, a high‐quality moiré graphene/MoS_2_ heterostructure was fabricated with the assistance of TPN and a Cu substrate. The preparation process of graphene/TMDCs heterostructures in the abovementioned case enhanced the adhesion of the interface, thereby significantly improving the electrical performance of the device based on this heterostructure. However, TPN residue may exist in the interface between graphene and MoS_2_, which affects the quality of the moiré graphene/MoS_2_ heterostructure.

Additionally, it is also possible to fabricate MoS_2_ after the growth of graphene (Figure 1i).^[^ ^55^ ^]^ Under the hydrogen‐containing condition, the oxidation of the graphene that is pregrown on the silicon is effectively avoided. Moreover, the hydrogen modifies the nucleation of MoS_2_ to directly form a large‐area monolayer MoS_2_ film on graphene. This method can mitigate the limitation of the residue and realize rapid preparation, which enables a convenient method to create lager‐area graphene‐based moiré heterostructures. However, the inability to precisely control the twist angle may considerably hinder the realization of graphene‐based moiré heterostructures. Thus, it is essential to develop other methods to prepare graphene‐based moiré heterostructures with a specific twist angle.

### 2.2 Assembling Monolayer 2D Materials

**Figure 2:**
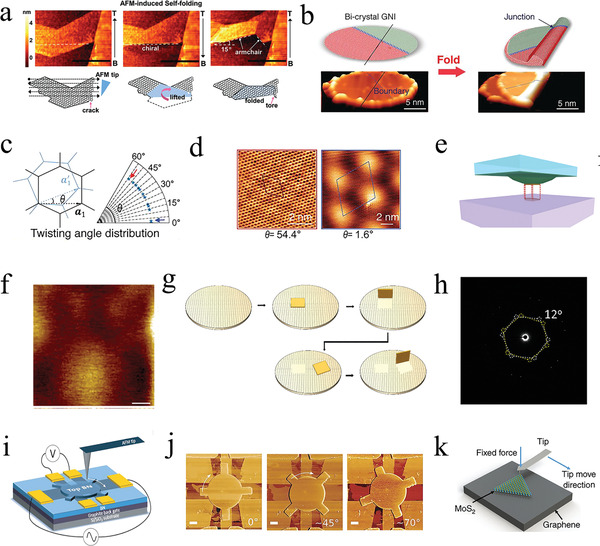
The graphene‐based moiré heterostructures by assembling monolayer 2D materials. a) AFM images and cartoons of self‐folding process using monolayer graphene. Reproduced with permission.^[^ ^63^ ^]^ Copyright 2018, Wiley‐VCH. b) Schematic image (top) and STM image (bottom) before and after folding monolayer graphene process. c) Schematic image of the tBLG with the twist angle in the range of 0° to 60°. d) STM images of the tBLG under the condition of twist angle *θ* = 54.4° and *θ* = 1.6°. b‐d) Reproduced with permission.^[^ ^64^ ^]^ Copyright 2019, American Association for the Advancement of Science. e) Schematic image of the hemispherical handle substrate. f) STM topography of the bilayer graphene. e,f) Reproduced with permission.^[^ ^65^ ^]^ Copyright 2016, American Chemical Society. g) Optical image of the pick and place monolayer graphene. h) The SAED of tBLG with twist angle of 12°. g,h) Reproduced under the terms of the Creative Commons CC‐BY license.^[^ ^66^ ^]^ Copyright 2019, The Authors. Published by MDPI. i) Schematic image of the experimental setup for twist angle regulation. j) AFM images of the moiré graphene/*h*‐BN heterostructures with three different orientations. i,j) Reproduced with permission.^[^ ^67^ ^]^ Copyright 2018, American Association for the Advancement of Science. k) Schematic image of AFM‐tip manipulation technique. Reproduced under the terms of the Creative Commons CC‐BY license.^[^ ^9^ ^]^ Copyright 2018, The Authors. Published by Springer Nature.

Using the in situ CVD growth method enables the convenient preparation of graphene‐based moiré heterostructures, which benefits large‐scale fabrication and application; however, there are a series of shortcomings regarding this method, such as the uncontrolled layers and twist angle. Compared with in situ CVD growth, assembling monolayer 2D materials enables precise control of the twist angle and layers, which assists research on physical properties. Thus, the simple folding^[^ ^60^ ^]^ or stacking^[^ ^61^, ^62^ ^]^ of exfoliated graphene flakes was typically used. In the folding process using an atomic force microscope (AFM) tip, the folding line selection is arbitrary and relies on whether there are cracks in the graphene sheet (**Figure** **2**a).^[^ ^63^ ^]^ Similarly, the folding process is also controlled using a scanning tunneling microscope (STM) tip (Figure 2b).^[^ ^64^ ^]^ The twist angle may be controlled precisely from 0° to 60° with an accuracy of 0.1° (Figure 2c), which realizes the regulation of a moiré superlattice (Figure 2d). The folding method has high accuracy; however, it is constrained to the preparation of tBLG and multilayer graphene and is unsuitable for graphene/*h*‐BN and graphene/TMDCs heterostructures.

To achieve a more extensive preparation of heterostructures and improve the accuracy of the twist angle, a facile tBLG fabrication method was introduced, which provides better tuning for the twisted graphene fabrication.^[^ ^65^ ^]^ A Bernal‐stacked BLG was accurately manufactured using transfers of MLG flakes to illustrate the realization of the rotational alignment. A hemispherical, bulb‐like handle substrate was fabricated using a transparent base substrate, such as glass or a planar polydimethylsiloxane (PDMS) mold, and an epoxy or PDMS droplet deposited onto the substrate to improve the adhesion (Figure 2e). The reason why PDMS was used as the graphene‐supporting layer is its flexibility, robustness, adhesion to samples, and transparency.^[^ ^68^ ^]^ The STM topography of the bilayer region of the sample exhibited no moiré patterns, which indicates that the two graphene layers were perfectly aligned and in agreement with Bernal stacking (Figure 2f). Additionally, this method depends only on the metallic alignment marks on the substrate to tune the rotational alignment, thereby providing a feasible method to prepare twisted graphene.

A facile pick‐and‐place method was introduced to fabricate high‐quality, precise, and uniform graphene‐based moiré heterostructures (Figure 2g). This method takes advantage of the different adhesions between the interfaces to selectively pick up the graphene.^[^ ^66^ ^]^ First, the single‐crystal MLG was prepared on a Ge (110) wafer, and a gold pattern was then fabricated on the as‐grown MLG. The most important factor was that annealing in H_2_ could reduce the adhesion between graphene and the Ge substrate. Thus, the graphene flake was picked up facilely, and tBLG was precisely fabricated by stacking single‐layer graphene. The selected area electron diffraction pattern of the tBLG exhibited a twist angle of 12°. This result indicates that the pick‐and‐place method is feasible (Figure 2h). In addition, using this method minimizes interlayer residues and precisely controls the twist angle, which may improve the performance of the twisted graphene‐based device.

The most difficult aspect in the preparation of moiré graphene/*h*‐BN and graphene/TMDCs heterostructures is still the precise control of the twist angle, which the CVD method cannot solve. To address this issue, a new method provided a means to achieve dynamic tunability of the twist angle.^[^ ^67^ ^]^ From the schematic illustration of the device used to tune the twist angle (Figure 2i), the top *h*‐BN flake was transferred to the top of the graphene, and this top *h*‐BN could then be freely rotated and translated using an AFM, owing to the weak vdW forces between graphene and *h*‐BN. This device realized precise tuning of the twist angle with an accuracy of 0.2° (Figure 2j), thereby providing a platform for the preparation of graphene‐based moiré heterostructures.

Similar to the fabrication of graphene/*h*‐BN heterostructures, graphene/MoS_2_ heterostructures were fabricated with a similar size and preferred orientations via the AFM‐tip manipulation technique to realize the tuning of the twist angle between the TMDCs and graphene (Figure 2k).^[^ ^9^ ^]^ The deterministic twist angles were controlled by regulating the tip movement direction and length. This method can achieve precise control of the twist angle. However, many shortcomings, such as the introduction of impurities, wrinkles, and destruction during the transfer process, may also occur; thus, the preparation of graphene‐based moiré heterostructures requires further study.

## 3 Enhancing Quality of Graphene‐Based Moiré Heterostructures

As mentioned previously, a series of preparation methods for graphene‐based moiré heterostructures were introduced. **Table** **1** systematically summarizes the comparison of different preparation methods used for graphene‐based moiré heterostructures in terms of their transfer methods, twist angle, flake size, and growth temperature. Among these methods, the most applicable and fundamental method is stacking MLG.^[^ ^60^ ^]^ Clearly, this method inevitably introduces at the transfer process. Therefore, it is important to optimize the transfer process to improve the quality of graphene flakes, which can promote the development of graphene‐based moiré heterostructures.

**Table 1:** The comparison of different preparation methods of graphene‐based moiré heterostructures

Preparation methods		Heterostructures	Transfer methods	Twist angle	Flake size	Growth temperature	Ref.
In situ growth	1. Epitaxial growth	a) tBLG	Wet transfer	0° and 30°	≈20 µm	1030 °C	[18]
	2. CVD	a) ABA‐TLG	None	None	≈60 µm	1050 °C	[44]
		b) MLG, AB‐BLG, ABA‐TLG, ABCA‐ tetralayer graphene	Dry transfer	None	≈30 µm	1075 °C	[45]
		c) Graphene/MoS_2_	None	None	None	900 °C	[54]
Assem‐bling	1. Folding	a) tBLG	Dry transfer	Predefined	None	None	[63]
		b) tBLG	Dry transfer	0–60°	None	None	[64]
	2. Stacking	a) Bernal‐BLG	Dry transfer	0°	None	None	[65]
		b) tBLG, twisted multilayer graphene	Dry transfer	Predefined	None	None	[66]
		c) Graphene/*h*‐BN	Dry transfer	Predefined	None	None	[67]

Moreover, improving the cleanliness of the graphene surface may further enhance the graphene surface state and reduce the impact of environmental factors.^[^ ^69^ ^]^ Introducing super clean graphene into the preparation of graphene‐based moiré heterostructures is considered to be favorable.

### 3.1 Optimizing Transfer Process

The performance of large‐area MLG grown using CVD is typically limited by the transfer‐process‐induced polymer residue. That is, the separation of graphene film from metal substrates (e.g., Cu) is an essential and crucial step, which, to a large extent, determines the quality of the transferred graphene. Consequently, the transfer process is crucial for obtaining high‐quality graphene‐based moiré heterostructures to widen its application. After a decade of research on graphene transfer, the transfer method is now divided into dry and wet transfer; both methods have their own advantages and disadvantages. Next, we introduce these methods in detail.

**Figure 3:**
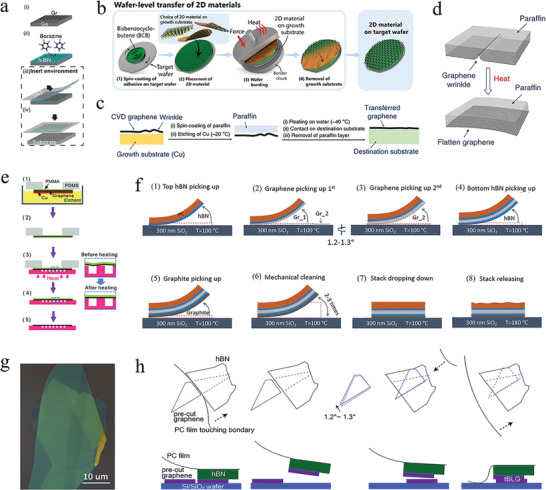
Optimizing the transfer process of the 2D materials. a) Schematic illustration of the dry transfer process of graphene film. Reproduced with permission.^[^ ^70^ ^]^ Copyright 2019, American Chemical Society. b) Schematic illustration of wafer‐level transfer of the 2D materials. Reproduced under the terms of the Creative Commons CC‐BY license.^[^ ^75^ ^]^ Copyright 2021, The Authors. Published by Springer Nature. c) Schematic illustration of the paraffin‐assisted graphene transfer process. d) Schematic illustration showing the effect of paraffin's thermal expansion on graphene wrinkle. Reproduced under the terms of the Creative Commons CC‐BY license.^[^ ^76^ ^]^ Copyright 2019, The Authors. Published by Springer Nature. e) Schematic illustration of graphene transfer process from Cu substrate to shallow depressions. Reproduced with permission.^[^ ^78^ ^]^ Copyright 2011, American Chemical Society. f) Schematic illustration of the stacking process to fabricate tBLG. g) Optical image of the tBLG after mechanical cleaning. f,g) Reproduced with permission.^[^ ^21^ ^]^ Copyright 2019, Springer Nature. h) Schematic illustration of the fabrication process for tBLG encapsulated by *h*‐BN flake. Reproduced with permission.^[^ ^79^ ^]^ Copyright 2020, Springer Nature.

Dry transfer is a versatile approach to realize the target materials exfoliated from the growth substrate to the target substrate. This method does not destroy the in‐plane covalent bonds, thereby minimizing the defect in target materials. For example, Yang et al. used a thermal‐release tape (TRT) to transfer graphene from a Ge (110) substrate to different substrates, owing to the weak interaction between graphene and the Ge (110) substrate.^[^ ^70^ ^]^ The transfer process is illustrated as follows (**Figure** **3**a). First, a graphene film was grown on a Ge (110) substrate via CVD. Subsequently, an *h*‐BN carrier film was prepared on the graphene surface. Then, the graphene/*h*‐BN was exfoliated via TRT and finally transferred to the target substrate. Although this method effectively avoids impurity residues and interface damage, the interaction between the target materials and carrier film must be larger than the binding force between graphene and the substrate, which results in the limited selection of substrates. The graphene films grown on typical substrates, such as Cu and SiC, do not meet this condition, which limiting the application of dry transfer. Thus, wet transfer has higher universality.

In the wet transfer process, the most widely used support layer is still polymethylmethacrylate (PMMA). However, the PMMA‐assisted process engenders some issues that severely degrade the quality of graphene: polymer contamination and graphene wrinkling.^[^ ^71^ ^]^ To address these issues, support layers made by other polymers have been attempted.^[^ ^72^, ^73^, ^74^ ^]^ For example, the adhesive bisbenzocyclobutene (BCB) benefited the transfer process of 2D materials.^[^ ^75^ ^]^ The transfer process from the growth substrate to the target wafer is depicted as follows (Figure 3b). First, BCB was spin‐coated onto the target wafer as an adhesive, and the 2D material was subsequently placed on top of the wafer. Then, heating and squeezing were performed to form a stable bond between the 2D material and target wafer substrate. Lastly, the growth substrate was removed by etching or other methods. This wet transfer technique is available to large‐scale transfer; however, the polymer residue may also be an obstacle. Similarly, paraffin is an excellent support layer, owing to its good thermal properties, low chemical reactivity, and weak interaction force.^[^ ^76^ ^]^ From the transfer process (Figure 3c), multilayer graphene film was synthesized on Cu foil, and the transfer support layer of paraffin wax was then spin‐coated on the as‐grown graphene, which was followed by etching the Cu substrate.^[^ ^77^ ^]^ After being rinsed with water several times, the sample was transferred to 40 °C water to minimize wrinkling by taking advantage of the high thermal expansion of paraffin. Finally, the transfer process was accomplished by scooping up the residual water and removing the paraffin layer. The MLG transferred onto flat substrates using this method has fewer cracks and tears (Figure 3d). However, the wet transfer techniques inevitably trap liquid inside the sample.

Suk et al. proposed a novel transfer technique to improve the transfer of CVD‐derived MLG grown on a copper substrate onto shallow wells to avoid trapping water and decrease the destruction when removing the support layer (Figure 3e).^[^ ^78^ ^]^ The copper substrate had a rough surface during the annealing and growth process at a high temperature; therefore, the transferred graphene supported by the polymer layer did not lie flat on the target substrate after removing the copper foil. Small gaps were generated in the interface, which caused an incomplete contact between the graphene and target substrate and unavoidably caused cracks and tears when the polymer layer was removed. The shallow wells reduced the surface tension via heating to obtain high‐quality target materials.

Additionally, optimizing the process conditions also improves the quality of graphene and precisely controls the twist angle (Figure 3f).^[^ ^21^ ^]^ A “tear and stack” process was conducted to fabricate tBLG to decrease the inhomogeneity of the structure.^[^ ^80^ ^]^ However, the annealing process was not used to clean the sample and remove the trapped water bubbles between the two layers to avoid the relaxation of the twist angle. Instead, gradually rolling the top layer from one edge of the bottom layer resulted in bubble‐free tBLG, and it released the local strain. Thus, the cleaning process significantly reduced the content of water bubbles (Figure 3g). Moreover, the transfer and stack process were performed at 100 °C to avoid the uncertainty caused by thermal expansion. However, except that the final stacks were carried out at 180 °C, owing to the melting point of polycarbonate. Unlike the “tear and stack” process, a new method called “cut‐and‐stack” prevented unintentional strain forming during the stacking process.^[^ ^79^ ^]^ From the fabrication of tBLG (Figure 3h), the only notable pattern of differences was that the MLG was cut into two pieces through an AFM instead of tearing the graphene encapsulated by an *h*‐BN flake.^[^ ^24^ ^]^ This fabrication technique significantly improved the quality of the heterostructure with the inhomogeneity of a twist‐angle less than 0.05° on a ≈10 µm length scale. The optimization of the transfer process conditions provides a new idea for the preparation of graphene‐based moiré heterostructures.

### 3.2 Improving Graphene Quality

It is difficult to precisely control the twist angle using CVD‐derived graphene, and exfoliated graphene is limited by size and scaled preparation. Currently, graphene‐based moiré heterostructures are primarily prepared by stacking or folding MLG and other 2D materials. Graphene‐based moiré heterostructures need atomically clean interfaces because the surface cleanliness significantly affects the physical properties of graphene. However, contaminants are typically trapped in the interlayer, which is incompatible with the fabrication of high‐quality graphene‐based moiré heterostructures. That is, the ex situ preparation method and the progress of the modern semiconductor industry have put forward higher requirements for graphene quality.

Graphene is a 2D monolayer material, which has a considerably large surface area. Thus, surface contamination may significantly degrade its intrinsic properties and strongly hinder its application in surface regions. The surface contamination of CVD‐derived graphene films mainly results from intrinsic contamination during the CVD growth process (amorphous carbon), polymer residues inevitably introduced in the transfer processes, and airborne contaminants. Generally, it is effective to eliminate transfer‐related contamination via annealing,^[^ ^81^ ^]^ plasma treatment,^[^ ^82^ ^]^ and mechanical cleaning.^[^ ^83^ ^]^ However, these postgrowth methods cannot thoroughly mitigate surface and interface contamination. It is essential to produce superclean graphene films by eliminating the main contamination of surface amorphous carbon to improve the quality of graphene and provide a high‐quality twisted graphene surface.

**Figure 4:**
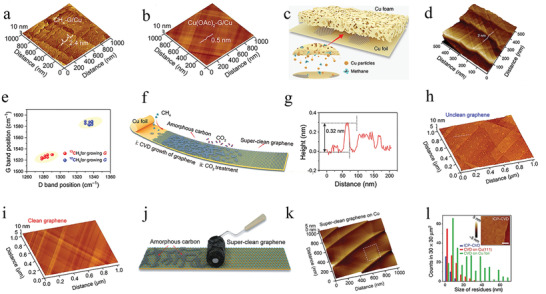
The methods of improving graphene quality. a) AFM image of CH_4_‐derived unclean graphene. b) AFM image of Cu(OAc)_2_‐derived superclean graphene. a,b) Reproduced with permission.^[^ ^69^ ^]^ Copyright 2019, American Chemical Society. c) Schematic illustration of the Cu foil‐foam architecture. d) AFM image of the clean graphene prepared on Cu foil‐foam architecture. e) TERS characterization of the D and G band positions from the contaminated regions of graphene grown by using normal methane (blue) and ^13^C‐labeled methane (red). c‐e) Reproduced under the terms of the Creative Commons CC‐BY license.^[^ ^22^ ^]^ Copyright 2019, The Authors. Published by Springer Nature. f) Schematic illustration of CO_2_‐assisted preparation of superclean graphene. g) Height profile of the unclean graphene marked in (h). h) AFM images of unclean graphene without CO_2_‐assisted. i) AFM images of superclean graphene with CO_2_‐assisted growth. f‐i) Reproduced with permission.^[^ ^84^ ^]^ Copyright 2019, Wiley‐VCH. j) Schematic illustration of cleaning graphene by the activated carbon‐coated lint roller. k) AFM images of superclean graphene after cleaning treatment. j,k) Reproduced with permission.^[^ ^85^ ^]^ Copyright 2019, Wiley‐VCH. l) Statistics of the residual nanoparticles on transferred graphene films after different growth processes. Inset shows AFM image of the ultraflat film with a scale bar of 1 µm. Reproduced with permission.^[^ ^86^ ^]^ Copyright 2020, Springer Nature.

Recently, the fabrication techniques of superclean graphene layers became well‐established. For example, superclean graphene could be easily prepared by changing the carbon source, substrate, or some postgrowth methods. Using a metal‐containing reagent, such as Cu(OAc)_2_, as the carbon source enables a large‐scale contamination‐free graphene film.^[^ ^69^ ^]^ During the CVD process, the copper‐containing carbon source significantly increased the Cu content in the gas phase, which ensured a sufficient supply of the carbon source. That is, the introduction of a copper‐containing carbon source suppressed the formation of amorphous carbon. From the AFM characterization, there was a lot of amorphous carbon on the CH_4_‐derived graphene surface. The average thickness was 2.4 nm (**Figure** **4**a), and no obvious amorphous carbon was observed on the Cu(OAc)_2_‐derived graphene surface (surface roughness < 0.5 nm) (Figure 4b). Moreover, designing copper substrate architecture improved the supply of the carbon source, which benefited the growth of superclean graphene.^[^ ^22^ ^]^ Using the substrate architecture via stacks of Cu foil and Cu foam (Figure 4c), the controlled preparation of superclean graphene films was realized (Figure 4d). Notably, the effective catalyst design, is the key parameter to the fabrication of superclean graphene, instead of the surface morphology of the substrate. In addition, a ^12^C/^13^C isotope‐labeling technique was employed to illustrate the origin of the surface amorphous carbon. In the tip‐enhanced Raman spectroscopy (TERS) characterization (Figure 4e), there was a difference in the contamination‐related peaks between the ^12^C‐CH_4_ and ^13^C‐CH_4_ derived graphene samples, which indicated that amorphous carbon was introduced during the high‐temperature CVD growth procedure.

A facile and scalable CVD method used to synthesize superclean graphene was introduced considering the difficulty in synthesizing large scale superclean graphene using the method discussed above.^[^ ^84^ ^]^ After the graphene growth, the weak oxidant CO_2_ was introduced to etch away the intrinsic contamination (Figure 4f). During the high‐temperature CVD growth process, amorphous carbon (≈0.3–3 nm thick) was formed on the graphene surface (Figure 4g), and the area ratio of the unclean surface was higher than 50% (Figure 4h). After the CO_2_ selective etchant at ≈500 °C, the amorphous carbon was removed (Figure 4i). Taking advantage of the softness of Cu foil to increase the contact between activated carbon and contaminants on graphene enabled the selective removal of surface contamination, which resulted in a superclean graphene surface (Figure 4j).^[^ ^85^ ^]^ Additionally, no clear contamination on the graphene surface was observed from the AFM characterization (Figure 4k).

Recently, a proton‐assisted CVD method (ICP‐CVD) was implemented to grow ultraflat graphene films that were wrinkle‐free, owing to the decoupling from the substrate.^[^ ^86^ ^]^ According to the statistical results of the nanoparticles (Figure 4l), there were almost no nanoparticles larger than 10 nm in the ICP‐CVD growth graphene films compared with other films, which indicated the realization of ultraflat graphene. Additionally, the ultraflat graphene easily removed surface residues that were introduced by the transfer processes.

In summary, the successful removal of amorphous carbon to fabricate superclean graphene can, to a large extent, reduce the polymer residues caused by the transfer process. Consequently, the graphene quality improves, and graphene‐based applications will also become more widespread, such as the fabrication of twisted graphene.

## 4 Topological Properties of Graphene‐Based Moiré Heterostructures

Many studies focused on developing and understanding graphene‐based moiré heterostructures comprising 2D materials with weak vdW forces.^[^ ^87^ ^]^ Looking beyond the field of the graphene‐based moiré heterostructures, research on graphene is extensive, and it tends to remain as one of the leading topics in condensed matter physics for many years. With rapid improvement in graphene fabrication techniques, graphene‐based moiré heterostructures should develop into a vast field, such as the field of many‐body physics. Additionally, many‐body interactions between electrons can induce various correlated phenomena, such as correlated insulator states,^[^ ^24^ ^]^ unconventional superconducting states,^[^ ^88^ ^]^ ferromagnetism,^[^ ^89^ ^]^ and the quantum Hall effect.^[^ ^5^, ^90^ ^]^ These special topological properties in graphene‐based moiré superlattices make the topological and correlated behaviors closely related. In this section, we introduce the topological properties of graphene‐based moiré heterostructures in detail, such as tBLG, tTLG, tDBG, twisted graphene/*h*‐BN, and graphene/TMDCs heterostructures.

### 4.1 Twisted Bilayer Graphene

Graphene‐based moiré heterostructures with a twist angle yield a structural moiré pattern, often inducing exotic properties.^[^ ^91^, ^92^ ^]^ tBLG is a typical case in which the lattice structure typically relaxes to attain a stable structure and then produces a moiré pattern.^[^ ^93^ ^]^ In particular, when the twist angle of tBLG was less than a few degrees, the Dirac cones of the two graphene layers were strongly hybridized by the moiré interlayer interactions, which introduced a moiré pattern with a long special period and significantly influenced the energy band structure.

In tBLG, the forming conditions of moiré patterns with long periods included rotation and the difference between the lattice vectors between the two layers of graphene.^[^ ^94^ ^]^ The following formula proposes the conditions for the formation of long period moiré pattern. The superlattice constant of the moiré supercell is $L_{s}=a/\sqrt{\eta^{2}+1-2\eta \cos\theta }$, where *a* is the lattice constant of MLG, *η* is the difference between the lattice vectors, and *θ* is the interlayer twist angle. It is clear that *L*_s_ is considerably larger than *a* under the conditions of *η* ≈ 1 and a relatively small *θ*. For tBLG, *η* = 1 and *a* = 2.46, that is, *L*_s_ = 2.46/2sin (*θ*/2).^[^ ^95^ ^]^ It is noted that the conditions for the formation of moiré patterns in the graphene‐based moiré heterostructures are similar.

**Figure 5:**
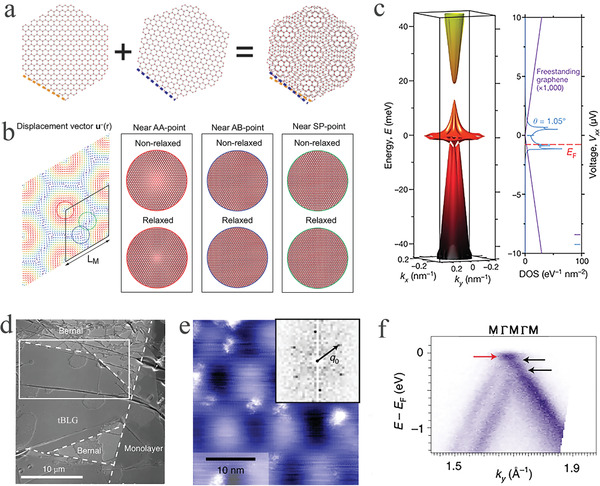
Topological properties of the tBLG. a) Schematic illustration of the moiré pattern formation process in the tBLG. Reproduced with permission.^[^ ^94^ ^]^ Copyright 2017, National Academy of Sciences. b) Outermost panel illustrated distribution of the displacement vector *u ^−^* (*r*) versus position in the tBLG with *θ* = 1.05°. Right side panels show local atomic structure near AA (AB or SP) stacked point before and after the relaxation. Reproduced with permission.^[^ ^23^ ^]^ Copyright 2017, American Physical Society. c) Schematic illustration of the band structure of the tBLG at *θ* = 1.05° and the DOS of this tBLG (blue) and two sheets of freestanding graphene (purple). Reproduced with permission.^[^ ^13^ ^]^ Copyright 2018, Springer Nature. d) Bright‐field LEEM image of the tBLG. e) STM topography of tBLG. Insert is the Fourier transform used for the determination of the twist angle. f) The spectral weight distribution of theoretical calculation at the mini‐Brillouin zone. d‐f) Reproduced with permission.^[^ ^96^ ^]^ Copyright 2021, Springer Nature.

Through the above introduction, the adjustment of the moiré pattern in tBLG was achieved by changing the twist angle. tBLG was precisely fabricated with twist angles smaller than 1° using a hemispherical handle substrate.^[^ ^94^ ^]^ Clearly, the moiré pattern formed owing to the small twist angle between the two graphene layers (**Figure** **5**a). Within a moiré unit cell, the two layers of atoms gradually changed between the AA and AB/BA (Bernal) regions, where AA exhibits the ideal overlapping of hexagons, and AB/BA represents the arrangement configurations, in which the A (B) sublattice was right above that of B (A). However, the two graphene layers of tBLG were not rigid, and the lattice structure could relax to change the atomic arrangement. To present the lattice relaxation in tBLG, the displacement vector *u ^−^* (*r*) depicted the formation of the AB/BA domain in tBLG as a function of the position.^[^ ^23^ ^]^ The result calculated through this function presented that, after a relaxation in the twist angle of 1.05°, the area of the AA domain had clearly decreased while the AB/BA domain expanded (Figure 5b). Moreover, an array of soliton boundaries gradually formed in the region of the saddle point (SP) and presented a triangular commensurate region structure with alternating AB and BA stacking domains.

Thus, a few layers of graphene is the only reported material with semiconductor properties that could be tuned by an external field.^[^ ^14^ ^]^ Moreover, the interlayer twist angle critically affected the electronic energy band structure.^[^ ^94^ ^]^ In particular, the “magic‐angle” tBLG with a relative angle near 1° was predicted to host flat bands, nearly 10 meV in width.^[^ ^23^ ^]^ As displayed in the schematic illustration of energy band structure and the density of states (DOS) (Figure 5c), the band energy of tBLG at *θ* = 1.05° was near charge neutrality at less than 15 meV, and the DOS presented the states of tBLG corresponding to the band energy (blue) and the freestanding graphene without interlayer interaction (purple, multiplied by 10^3^).^[^ ^13^ ^]^ The energy band structure of tBLG changed significantly, owing to the twist angle and interlayer vdW interaction. Additionally, a series of correlated phenomena were introduced because electron−electron interaction was dominant in this flat band system.^[^ ^97^, ^98^ ^]^ Thus, tBLG is a preeminent platform for investigating such a super flat band structure, which can be realized by stacking graphene layers and exerting a vertical displacement field (*D*).

The bandwidth of a flat band in twisted graphene is generally obtained from theoretical calculation.^[^ ^99^ ^]^ However, given the size of the unit cell in twisted graphene, it is demanding to systematically describe the energy band structure of twisted graphene. Thus, it is important to seek an effective method to test the existence of the flat band structure in graphene. Traditionally direct electronic structure measurements using ARPES detected the flat bands in the occupied states;^[^ ^100^ ^]^ however, it is challenging to precisely test the partially filled flat band, which is considered to be the cause of the correlated behavior of twisted graphene near the magic angle. To provide direct evidence for the abovementioned limitation, the band dispersion near charge neutrality in tBLG was directly mapped by combining imaging techniques of low‐energy electron microscopy (LEEM), STM, and ARPES.^[^ ^96^ ^]^ There are two main reasons behind the combing of LEEM and STM. First, the STM image of tBLG provided accurate information on the twist angle corresponding to the periodicities of the moiré superlattice, owing to the twist angle inhomogeneity caused by the absence of *h*‐BN encapsulation in tBLG (Figure 5d).^[^ ^101^ ^]^ However, the bright‐field LEEM image clearly revealed a large‐area flat region with several round features associated with the agglomerates of polymer residues (Figure 5e). After a thorough characterization of the twist angle by STM and ensuring the cleanliness of the surface by LEEM, the ARPES characterization reflecting the moiré superlattice was implemented. From the ARPES (Figure 5f), the dominant gaps of ≈100 meV are comparable to interlayer coupling, thereby proving the existence of a flat band structure from the perspective beyond theoretical calculations.

### 4.2 Twisted Trilayer Graphene

In graphene‐based moiré heterostructures, the topological properties depend on the number of graphene layers, and the external environment results from encapsulating the layers. The topological performance of tTLG is primarily affected by the number of graphene sheets. Different from tBLG, the “moiré superlattice” of tTLG generates “moiré of moiré” superlattices with a larger lattice scale than that of tBLG, owing to the increase in the number of graphene layers.^[^ ^102^ ^]^ Additionally, tTLG has more arrangements, such as ABA‐TLG,^[^ ^103^ ^]^ ABC‐TLG,^[^ ^24^ ^]^ twisted monolayer–bilayer graphene (tMBG),^[^ ^104^ ^]^ and magic‐angle twisted trilayer graphene (MATTG).^[^ ^105^ ^]^ These arrangements provide a tunable platform for the further research of topological and correlated properties.

**Figure 6:**
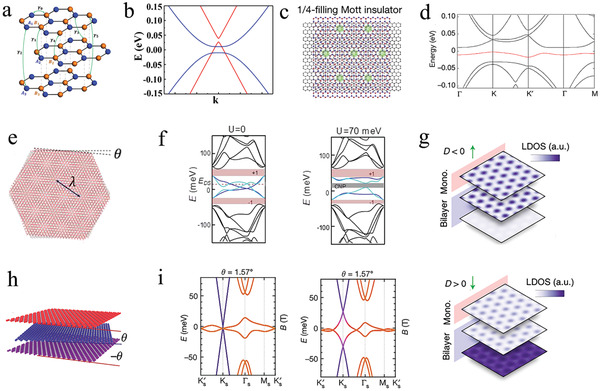
Topological properties of the tTLG. a) Schematic illustration of the ABA‐TLG. b) Calculated energy band structure of the TLG. a,b) Reproduced with permission.^[^ ^107^ ^]^ Copyright 2018, American Chemical Society. c) Schematic illustration of the triangular ABC‐TLG/*h*‐BN moiré superlattice and the 1/4‐filling Mott insulating state. d) The energy band structure of the ABC‐TLG with the bandgap of −20 meV. c,d) Reproduced with permission.^[^ ^14^ ^]^ Copyright 2019, Springer Nature. e) Schematic illustration of the moiré pattern by stacking MLG and BLG with a relative twist angle *θ*. *λ* is the wavelength of moiré pattern. f) Calculated energy band structures of the tMBG with *θ* ≈ 1.22° under *U* = 0 (left) and 70 meV (right). e,f) Reproduced with permission.^[^ ^108^ ^]^ Copyright 2021, Springer Nature. g) Calculated local density of states for each layer of the tMBG at full filling of the conduction band with *D* < 0 (left) and *D* > 0 (right). Reproduced with permission.^[^ ^104^ ^]^ Copyright 2021, Springer Nature. h) Schematic illustration of the MATTG. i) The energy band structures of the MATTG at zero (left) and finite (right) *D*/*ε*_0_ = 0.2 V nm^−1^. h,i) Reproduced with permission.^[^ ^11^ ^]^ Copyright 2021, Springer Nature.

From the moiré patterns and energy band structures of these four different arrangements, ABA‐TLG enhanced the electron−electron interaction, owing to the existence of strong trigonal warping in the absence of a magnetic field.^[^ ^106^ ^]^ From the lattice schematic (**Figure** **6**a) and energy band structure (Figure 6b) of Bernal‐stack ABA‐TLG, it was clear that there were MLG‐ and BLG‐like energy bands.^[^ ^107^ ^]^ Notably, different from ABA‐TLG, the moiré superlattice of ABC‐TLG offered a tunable Hubbard system to determine the mechanism of high‐temperature superconductivity.^[^ ^14^ ^]^ This system exhibited a series of triangular moiré superlattice and Mott insulator states upon applying a vertical displacement, which corresponded to a 1/4 filling of the superlattice unite cell (Figure 6c). Consisted with the moiré superlattice, the calculated energy band structure with a potential energy difference was 2∆ = −20 meV under the condition of *D* = −0.4 V nm^−1^ (Figure 6d). Additionally, the first hole miniband (highlighted in red) was superflat with a bandwidth of 11.7 meV and was separated from other bands over 10 meV. This special flat band structure of the ABC‐TLG heterostructures offered a series of attractive electron−electron correlations such as tunable superconductivity,^[^ ^88^ ^]^ a correlated insulating state, metal, correlated resistive state, magnetism,^[^ ^89^ ^]^ and departures from Fermi liquid behavior in the metallic state.^[^ ^109^ ^]^

For tMBG, the twist angle becomes the key parameter of the lattice structure (Figure 6e) because tMBG is composed of rotated sheets of MLG and a Bernal‐stacked BLG.^[^ ^108^ ^]^ The bandgaps at the charge neutrality point (CNP) increased with the application of energy U (Figure 6f), which indicates the appearance of a correlated insulating state. Moreover, the correlated states approximately resemble tDBG when the field points from MLG to BLG (*D* > 0) because tMBG has low symmetry. However, the correlated states resemble tBLG at *D* < 0 (Figure 6g).^[^ ^104^ ^]^ Based on the above studies for tBLG and TLG, an MATTG containing three pieces of graphene monolayers twisted by *θ* and −*θ* was fabricated, in which the twist angle could be tuned independently (Figure 6h).^[^ ^11^ ^]^ According to the theoretical calculation, the magic angle in MATTG was 1.6°. The energy band structures of the MATTG near the magic angle with (left panel) and without (right panel) *D* are depicted (Figure 6i). Upon applying *D*, the flat bands and Dirac bands without an energy gap hybridized. That is, the bandwidth and strength of the interaction of the flat bands was tuned by the magnitude of *D*. Moreover, a similar system was prepared with the twist angle within the scale of 2°–3°.^[^ ^110^ ^]^ This platform exhibited a semimetallic state, which was different from tBLG at full filling of the supercell for the first time in the higher‐order moiré flat bands. This result accelerated future research on moiré correlated states and topological properties.^[^ ^21^ ^]^

As mentioned in the previous sections, the carrier concentration and bandwidth of the twisted graphene were controlled independently through an external field.^[^ ^111^, ^112^ ^]^ Herein, we consider the example of a Hall bar geometry device based on ABC‐TLG to introduce the regulation of the energy band structure.^[^ ^14^ ^]^ The bandwidth and charge concentration are tuned by $D=\frac{1}{2}\left(D_{b}+D_{t}\right)$ and *n*  = (*D*_b_ + *D*_t_)/*e* , where $D_{b}=+\varepsilon_{b}\left(V_{b}-V_{b}^{0}\right)/d_{t}$ and $D_{t}=-\varepsilon_{t}\left(V_{t}-V_{t}^{0}\right)/d_{t}$ are controlled through the bottom and top voltages, respectively. Here, *ε*_b_ and *ε*_t_ are the relative dielectric constants of the bottom dielectric layer with a thickness of *d*_b_ and the top dielectric layer with a thickness of *d*_t_, respectively. In this device, the dielectric layers were composed of *h*‐BN with *ε* ≈ 3.9. These examples further illustrate the adjustable characteristic of the carrier concentration and bandwidth in graphene‐based moiré heterostructures.

### 4.3 Twisted Double Bilayer Graphene

**Figure 7:**
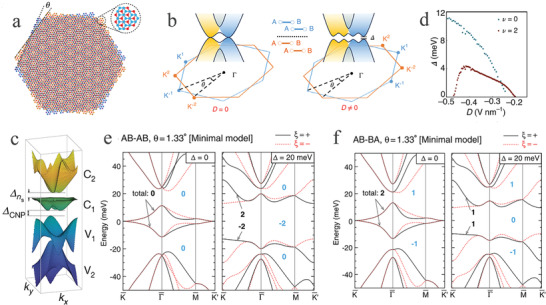
Topological properties of the tDBG. a) The moiré superlattice of the tDBG. Reproduced with permission.^[^ ^10^ ^]^ Copyright 2020, Springer Nature. b) Schematic illustration of the electrical tuning mechanism. Reproduced with permission.^[^ ^114^ ^]^ Copyright 2020, Springer Nature. c) Calculated energy band structure for *θ* = 1.33° tDBG at an optimal *D*. Reproduced with permission.^[^ ^16^ ^]^ Copyright 2020, Springer Nature. d) Energy gaps of the CNP (*ν* = 0) and the CI state at *ν* = 2 as a function of *D*. Reproduced with permission.^[^ ^115^ ^]^ Copyright 2021, Springer Nature. e,f) Energy band structures of the twisted AB–AB (e) and AB–BA (f) tDBG at the twist angle of 1.33° with Δ = 0 and 20 meV. e,f) Reproduced with permission.^[^ ^116^ ^]^ Copyright 2019, American Chemical Society.

With the continuous research on the correlated phenomena of tBLG and tTLG, tDBG has gradually become a tunable platform to study and observe similar exotic behaviors. On the one hand, a series of correlated states have been achieved in tBLG under the conditions of *θ* > 1.2° and a high external field;^[^ ^88^ ^]^ however, the bandwidth of the flat band could not be tuned by *D*.^[^ ^94^ ^]^ On the other hand, the observation of correlated behaviors has been realized in ABC‐TLG by applying *D*.^[^ ^14^ ^]^ The bandgap of Bernal (AB)‐stacked BLG could be opened under an external field;^[^ ^113^ ^]^ therefore, tDBG was very likely to exhibit a flat band and correlated phenomena. Thus, extensive research on tDBG has been conducted recently based on electron−electron interaction‐driven correlated phases. tDBG was not composed of MLG, but a pair of exfoliated AB‐stacked BLG with a twist angle of *θ* (**Figure** **7**a).^[^ ^10^ ^]^ The energy band structures of tDBG and tBLG nearly resemble each other under the condition of considering the AB‐stacked BLG as a whole. Unlike tBLG, the energy band structure of tDBG could be tuned by exerting *D*, whereas *D* cannot open an energy gap of tBLG at the charge neutral point, owing to the C_2_T symmetry and strong interlayer interaction. When the external field was applied, the bandgap was opened up at charge neutrality, and the conduction band flattened with increasing |*D*| (Figure 7b).^[^ ^114^ ^]^ A shallow Mexican‐hat‐shaped energy dispersion vividly formed at a finite *D* in tDBG (Figure 7c).^[^ ^16^ ^]^ Additionally, the regulation of the energy band structure through *D*, to a large extent, affects the correlated behaviors. For example, the bandgap began to appear at |*D*| = 0.25 V nm^−1^, and it increased continually with an increase in |*D*| at charge neutrality (*ν* = 0), thereby indicating that the correlated insulating state existed at |*D*| ≳ 0.25 V nm^−1^ (Figure 7d).^[^ ^115^ ^]^ Moreover, the correlated insulating state emerged in the |*D*| value range of 0.20–0.47 V nm^−1^ at *ν* = 2 using thermal activation measurements, which further verifies that the energy band structure and correlated states could be tuned by an external field.

Koshino studied the energy band structures and topological properties of AB–AB and AB–BA considering the difference in arrangement between the two systems.^[^ ^116^ ^]^ The energy band structures of AB–AB and AB–BA arrangements were similar; however, their topological properties were completely different. Judging from the minimal‐model energy band structures of AB–AB (Figure 7e) and AB–BA (Figure 7f) tDBG with a twist angle of 1.33°, one can observe that the crucial difference between the two arrangements is the Chern number. Under the condition of Δ = 0, AB–AB tDBG was a trivial insulator, whereas AB–BA tDBG was a valley Hall insulator with a nonzero Chern number, owing the absence of a symmetry constraint. Additionally, by adjusting *D* to tune Δ = 20 meV, the Chern numbers of the two systems all altered to nonzero values because the energy gap at the charge neutrality point was opened by Δ.

### 4.4 Twisted Graphene/*h*‐BN Heterostructures

**Figure 8:**
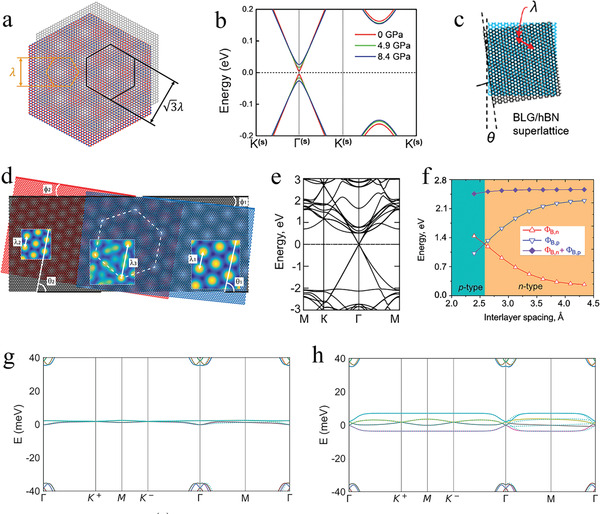
Topological properties of the twisted graphene/X (X = *h*‐BN, TMDCs) heterostructures. a) Schematic illustration of the moiré superlattice in graphene/*h*‐BN heterostructure. Reproduced with permission.^[^ ^120^ ^]^ Copyright 2017, American Chemical Society. b) Calculated energy band structure of the ≈1.8° graphene/*h*‐BN heterostructure at 0, 4.9, and 8.4 GPa. Reproduced with permission.^[^ ^25^ ^]^ Copyright 2020, American Chemical Society. c) Schematic illustration of the BLG/*h*‐BN superlattice. Reproduced with permission.^[^ ^121^ ^]^ Copyright 2020, American Chemical Society. d) Schematic illustration of three different moiré superlattices formed in the *h*‐BN/graphene/*h*‐BN heterostructure. Reproduced with permission.^[^ ^122^ ^]^ Copyright 2019, American Chemical Society. e) Calculated band structure of the graphene/GaS heterostructure. f) The Schottky barrier height in the graphene/GaS heterostructure as a function of the interlayer spacing. e,f) Reproduced with permission.^[^ ^123^ ^]^ Copyright 2018, AIP publishing. g,h) Energy band structures along a high symmetry line of the BM Hamiltonian with a Rashba SOC term. The twist angle is *θ* = 1.08°, and $\lambda_{R}^{+}$ = 16 meV (g), and $\lambda_{R}^{-}$ = 16 meV (h). g,h) Reproduced with permission.^[^ ^124^ ^]^ Copyright 2020, American Chemical Society.

*h*‐BN was introduced as a substrate, owing to the flat surface of *h*‐BN and the similar lattice structure between *h*‐BN and graphene.^[^ ^117^, ^118^ ^]^ As an interesting phenomenon, bandgaps were sometimes observed in graphene/*h*‐BN, which has attracted broad attention.^[^ ^119^ ^]^ In particular, the crystal lattices of graphene/*h*‐BN introduced a periodic moiré pattern, and the moiré wavelength was directly correlated to the twist angle, which facilitated new opportunities for exploring the correlated phenomena.^[^ ^5^ ^]^ The forming of the moiré graphene/*h*‐BN heterostructures fundamentally modified the energy band structure of graphene. When graphene was stacked on *h*‐BN, secondary Dirac points (the orange hexagon marking) appeared, while a set of tertiary Dirac points (the black hexagon marking) was observed under finite magnetic field conditions (**Figure** **8**a).^[^ ^120^ ^]^ These special tertiary points primarily benefited from the twist angle between the graphene and *h*‐BN. However, the effects were weakened, and they even disappeared at a large twist angle. Thus, an external regulation was required. In the ∼1.8° twisted graphene/*h*‐BN system near the primary Dirac point (PDP) (Figure 8b), the moiré wavelength became shorter and the in‐plane structural deformation was insignificant, which resulted in a smaller PDP gap as the twist angle increased.^[^ ^25^ ^]^ Upon applying strain, the spacing between layers diminished, and the in‐plane relaxation was enhanced, thereby leading to larger PDP gaps.

In addition to the above‐mentioned moiré heterostructures composed of MLG and *h*‐BN, there are other graphene/*h*‐BN heterostructures, such as BLG/*h*‐BN,^[^ ^121^ ^]^ TLG/*h*‐BN,^[^ ^125^ ^]^ and *h*‐BN/graphene/*h*‐BN.^[^ ^122^ ^]^ The moiré pattern of the BLG/*h*‐BN heterostructures with wavelength *λ* and twist angle *θ* illustrated the alignment between graphene and *h*‐BN (Figure 8c).^[^ ^121^ ^]^ The introduction of a BLG/*h*‐BN heterostructure led to an intensive investigation examine of single‐carrier transport, which laid the foundations for exploring the relationship between single‐electron transport and correlated behaviors in 2D quantum materials. In the nearly aligned ABC‐TLG/*h*‐BN heterostructures, the electron−electron interactions resulted in correlated phases. Furthermore, the superlattice flat bands of ABC‐TLG/*h*‐BN had a significant advantage compared to tBLG because they were less sensitive to the twist angle, which provided a promising platform for the research of topological and correlated phenomena. Additionally, fully *h*‐BN‐encapsulated graphene formed a moiré *h*‐BN/graphene/*h*‐BN heterostructure.^[^ ^122^ ^]^ In this system, diverse moiré patterns formed via the top *h*‐BN/graphene, the bottom *h*‐BN/graphene, and *h*‐BN/graphene/*h*‐BN (Figure 8d). The wavelength of the *h*‐BN/graphene/*h*‐BN system was larger than the maximum moiré periods of graphene/*h*‐BN, which enabled the intensive research of moiré graphene band structure.

### 4.5 Twisted Graphene/TMDCs Heterostructures

The graphene‐based moiré heterostructures with twisted graphene/TMDCs provide a novel platform, owing to their remarkable properties, such as spin–orbit coupling^[^ ^17^ ^]^ and electrical and thermal conductivity.^[^ ^40^ ^]^ Additionally, the form of twisted graphene/TMDCs heterostructures is very rich. Not only is the number of graphene layers adjustable to be either monolayer or bilayer, but TMDCs also consist of diverse transition metals and chalcogenide. In addition, the layers of TMDCs can be altered as well as graphene. Thus, twisted graphene/TMDCs heterostructures have wide potential for future research. Next, we specifically introduce MLG/TMDCs, BLG/TMDCs, and multilayer graphene/TMDCs heterostructures.

In MLG/GaS heterostructures, the intrinsic properties of isolated graphene and GaS were reserved, owing to the weak interaction in the interface.^[^ ^123^ ^]^ From the calculated energy band structure (Figure 8e), the linear dispersion relation in graphene and the bandgap of 2.55 eV in GaS further proved the weak vdW interaction between graphene and GaS. In addition, the Schottky barrier formed due to the Fermi level of the MLG/GaS heterostructure situated in the bandgap area of GaS. Thus, an effective method of applying vertical strain to diminish the interlayer space was introduced to regulate the electronic properties and Schottky barrier height. It is noted that the Schottky barrier height could be tuned efficaciously by changing the interlayer space (Figure 8f).^[^ ^123^ ^]^ Similarly, the Schottky barrier in the graphene/MoS_2_ heterostructures can also be adjusted by the gate, which enables research on spintronic properties because the controllable Schottky barrier contributes to the control of the spin parameters.^[^ ^17^ ^]^

For tBLG, the band structure comprised eight bands near the magic angle with a small bandwidth, and the flat bands were significantly changed once the tBLG was placed on a TMDCs substrate, owing to the spin–orbit coupling introduced by the proximity effect.^[^ ^124^ ^]^ Similar to the effect of *h*‐BN on the band structures of graphene,^[^ ^126^ ^]^ small external impacts resulting from the substrate altered the phase diagram of twisted graphene. The band structures were visibly altered, owing to the opposite spin–orbit coupling (Figure 8g,h).^[^ ^124^ ^]^ Of course, not all the external conditions strongly impact the graphene‐based moiré heterostructures, such as the stacking order and the twist angle between graphene and other 2D materials. For example, the graphene/MoS_2_ heterostructures with multilayer MoS_2_ and MLG indicated that layer‐breathing coupling in the graphene‐based moiré heterostructures resulted in new lattice vibration modes, which favored the understanding of graphene‐based moiré heterostructures for further electronic and photonic applications.^[^ ^127^ ^]^

## 5 Correlated Physical Performances in Graphene‐Based Moiré Heterostructures

Benefiting from the topological properties mentioned above, graphene‐based moiré heterostructures exhibit a series of unique physical properties. The observation of new physical phenomena in graphene‐based moiré heterostructures resulted from an unprecedented degree of control of the twist angle, strain, and dielectric environment. In this section, we introduce the factors that influence correlated physical performances and various unique physical properties in graphene‐based moiré heterostructures.

### 5.1 Factors that Influence Correlated Physical Properties

Studies on the correlated physical properties in graphene‐based moiré heterostructures have concentrated primarily on the twist angle, which has a crucial role in tuning and controlling physical properties, such as superconductivity, correlated insulating state, ferromagnetism, and quantum Hall states. This is owing to the tunable flat bands created by the hybridization between graphene layers^[^ ^128^ ^]^ and isolation from other parts of the energy spectrum by a large energy gap of almost 30 meV.^[^ ^109^, ^129^ ^]^ A nonuniform distribution of strain may lead to the geometrical deformation of the graphene‐based moiré superlattice, thereby leading to the introduction of a strong gauge potential to act as a pseudo magnetic field (PMF).^[^ ^130^ ^]^ In addition to the twist angle and strain in graphene‐based moiré heterostructures, the correlated states were also influenced by the dielectric environment, such as the quality of *h*‐BN, which exhibited similar crystal lattices to graphene.^[^ ^89^ ^]^ The following sections specifically introduce the influence factors that influence correlated performances in graphene‐based moiré heterostructures.

#### 5.1.1 Twist Angles

**Figure 9:**
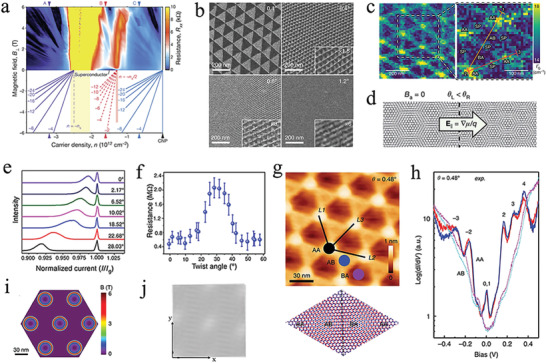
Influence of twist angle and strain in graphene‐based moiré heterostructures. a) Resistance versus *n* and *B*_⊥_ in the tBLG. The lower panel shows the Landau‐level structure deduced from the quantum oscillations. Reproduced with permission.^[^ ^13^ ^]^ Copyright 2018, Springer Nature. b) The dark‐field TEM images of the tBLG with various controlled twist angles. Reproduced with permission.^[^ ^134^ ^]^ Copyright 2019, Springer Nature. c) Nano‐Raman intensity of the 2D peak of tBLG. Reproduced with permission.^[^ ^135^ ^]^ Copyright 2021, Springer Nature. d) Schematic image of the MATBG sample with two connected regions of different twist angles, leading to an internal electric field *E*_||_. Reproduced with permission.^[^ ^101^ ^]^ Copyright 2020, Springer Nature. e) Normalized current distributions of the graphene/MoS_2_ heterostructures with various twist angles. f) Statistic resistances of the graphene/MoS_2_ heterostructures with different twist angles. e,f) Reproduced under the terms of the Creative Commons CC‐BY license.^[^ ^9^ ^]^ Copyright 2018, The Authors. Published by Springer Nature. g) STM topography image of the hexagonal moiré pattern (top panel) and the atomic model of the AA, AB, and BA stacking regions (bottom panel) of the tBLG. h) Logarithmic d*I*/d*V* spectra measured at the AA and AB regions. i) Calculated PMF. g‐i) Reproduced under the terms of the Creative Commons CC‐BY license.^[^ ^136^ ^]^ Copyright 2020, The Authors. Published by Springer Nature. j) STM image of the moiré pattern in the tBLG. Reproduced with permission.^[^ ^137^ ^]^ Copyright 2019, American Chemical Society.

The importance of the twist angle was realized owing to the discovery of superlattice Dirac points and the Hofstadter spectrum in graphene.^[^ ^3^, ^131^ ^]^ Under a magnetic field, the continuous band reorganized into a series of separate Landau levels in the 2D electronic system (**Figure** **9**a).^[^ ^13^ ^]^ Landau‐level structures with different filling factors were ascribed to the lattice mismatch, that is, the twist angle has become indispensable in the field of graphene‐based moiré heterostructures. Graphene‐based moiré heterostructures fabricated using different 2D materials offered an effective platform for solving many fundamental physics problems.^[^ ^132^ ^]^ As the foremost parameter, the twist angle between the different layers is critical in regulating the correlated performances of graphene‐based moiré heterostructures. Particularly, the regulation of the interlayer twist angle in graphene‐based moiré heterostructures has the ability to control the length of the moiré superlattice and the gap between its valence and conduction bands.^[^ ^13^, ^94^, ^133^ ^]^ Therefore, it is theoretically possible to prepare twisted devices, such as vertical field‐effect transistors and photodetectors, which may be tuned on‐demand by simply rotating the heterostructures. Indeed, many fascinating properties of the twist angle of graphene‐based moiré heterostructures have been researched such as unconventional superconductivity in tBLG,^[^ ^13^ ^]^ the gate‐tunable Mott insulator in TLG,^[^ ^24^ ^]^ tunable correlated states in tBLG,^[^ ^10^ ^]^ quantum transport in the graphene/*h*‐BN superlattice,^[^ ^67^ ^]^ and the spin Hall effect in graphene/MoS_2_ heterostructures.^[^ ^58^ ^]^ These correlated effects and the development of condensed matter physics based on graphene‐based moiré heterostructures require further study. An emerging field referred to as “twistronics” may realize novel electronic phenomena in graphene‐based moiré heterostructures by tuning the twist angle between successive layers.^[^ ^128^ ^]^

In addition, the lattice relaxation caused by the twist angle has an impact on the electronic structures by changing the interlayer and intralayer Hamiltonian. A tBLG with a twist angle in the range of 0° < *θ* < 4° exhibited the impact of the lattice relaxation on the atomic and electronic reconstruction at the vdW interface.^[^ ^134^ ^]^ The stacking order of tBLG was characterized using transmission electron microscopy (TEM) dark‐field imaging (Figure 9b). Additionally, the formation of the commensurate domains, which resulted from atomic and electronic reconstruction, was demonstrated. In the tBLG dark‐field image, a series of tBLG samples with different twist angles exhibited a tessellation. Particularly, tBLG with a 0.1° twist angle presented a triangular domain that alternated with the AB/BA domains, which are associated with the moiré pattern. The AB/BA domains observed at 0.1° separated by sharp mirror boundaries indicated the atomic and electronic reconstruction at the vdW interface of tBLG. Additionally, the atomic and electronic reconstruction could also be clearly distinguished from the nano‐Raman spectral image of tBLG (Figure 9c). ^[^ ^135^ ^]^ As the twist angle increased, the triangular domains became closer to one‐directional fringes, which indicated that the reconstruction strength increased gradually with decreasing *θ*. Thus, precise regulation and measurement of the twist angle has become particularly important.

Although the global *θ* could be precisely controlled in a narrow range of ≈0.1°^[^ ^12^, ^13^, ^21^, ^26^, ^88^, ^89^, ^138^ ^]^ and the correlated states could be observed down to 0.93°,^[^ ^139^ ^]^ it is difficult to obtain the distribution of the local twist angles. Consequently, the correlated states introduced by electron−electron interactions were suppressed because the kinetic energy gradually became dominant, and the flat bandwidth rapidly increased with the twist angle away from the magic angle.^[^ ^12^ ^]^ To obtain the relevant information on the local twist angles and understand their influence of the local twist angles on the energy band structures, one of the most possible reasons was proposed. The existence of defects caused the twist angle to relax and produce a twist angle gradient (Figure 9d), thereby forming a planar electric field in the sample. This significantly changed the quantum Hall state, thereby indicating that other correlated phases, such as magnetism and superconductivity in tBLG, may also undergo a fundamental transformation from the twist‐angle disorder.^[^ ^101^ ^]^

In addition to the physical phenomena introduced by the twist angle in the abovementioned graphene‐based moiré heterostructures, unraveling the effects of the twist angle in graphene/TMDCs heterostructures is of fundamental importance. In graphene/MoS_2_ heterostructures, the vertical conductivity was investigated via a conductive AFM scanning mode under various twist angle configurations.^[^ ^9^ ^]^ The current mapping was inverted into a statistical chart to quantitatively extract the conductivity (Figure 9e). It was clearly observed that the peak of the graphene/MoS_2_ heterostructures gradually moved apart to the graphene peak as the twist angle changed from 0° to 30°. From the calculated resistances of graphene/MoS_2_ heterostructures (Figure 9f), the vertical conductivities at a twist angle of 30° were modulated approximately four times higher than those at *θ* = 60° and *θ* = 0°. The twist‐angle‐dependent conductivities provided guidance by using graphene to reduce the contact resistance in semiconductors.

#### 5.1.2 Strain

The existence of the twist angle in tBLG introduced a regular moiré superlattice, however, a nonuniform distribution of strain^[^ ^130^ ^]^ led to the geometrical deformation of the graphene‐based moiré superlattice. Therefore, a strong gauge potential was introduced to act as the PMF. In particular, as the strain varied slightly, the Dirac cones of the two layers of graphene superlattice shifted to opposite directions, and a PMF was generated without breaking the time‐reversal symmetry of the crystal.^[^ ^140^, ^141^ ^]^ In addition, a large PMF^[^ ^142^ ^]^ with different shapes could be created by tuning the strain imposed on graphene, such as the strain arrays introduced by the thermal buckling transition,^[^ ^143^ ^]^ which was attractive for the management of valley spintronics in graphene.^[^ ^144^ ^]^ Thus, the studies on the PMF of the low‐angle tBLG provide a platform for the exploration of correlated physical phenomena in 2D vdW systems.

Indeed, the observation of the PMF in strained graphene in the shape of nanobubbles was realized using scanning tunneling microscopy/spectroscopy (STM/S),^[^ ^145^ ^]^ and the PMF derived in the tBLG was theoretically studied.^[^ ^146^, ^147^ ^]^ However, the experimental explorations into the evidence of the PMF in tBLG are still lacking.^[^ ^148^ ^]^ To address this problem, the pseudo‐Landau levels derived by the PMFs were first detected by combining the STM/S experiments and large‐scale tight‐binding calculations based on tBLG with small twist angles (*θ* < 1°).^[^ ^136^ ^]^ The STM topography image depicted hexagonal symmetry moiré patterns with a twist angle of 0.48° (Figure 9g). The characterization of STS provided information on the electronic properties of tBLG (Figure 9h). The AA domains revealed a series of peaks with an almost equal energy interval. These peaks were similar to the Landau levels of the tBLG under a strong external magnetic field. However, none of these characteristic peaks was obtained for the AB domains, which indicates that the PMF was generated in the AA regions.^[^ ^149^ ^]^ Additionally, to illustrate the agreement between the experimental observation and theoretical calculations, the calculation of the PMF magnitude is presented (Figure 9i). The calculations show that the PMFs were not uniform, but they formed a ring‐structured vortex lattice centered on the AA domains. Moreover, PMF was absent in the AB regions, which were in accord with the experimental characterizations.

The strain in graphene‐based moiré heterostructures acted as an effective parameter to tune its exotic properties, such as spin and valley properties.^[^ ^150^ ^]^ In particular, the strain between graphene layers provided an in situ method to create and modify moiré patterns via a piezoelectric substrate.^[^ ^140^ ^]^ Then, the effect of strain was systematically studied on moiré band structures for tBLG using a homobilayer system.^[^ ^137^ ^]^ Taking the twist angle of 1.05° as an example, the moiré pattern changed from dots to an elliptical shape, owing to the uniaxial strain with a strain magnitude *ɛ* = 0.7% and a strain direction *ϕ* = 0° (Figure 9j).^[^ ^137^ ^]^ Compared with the unstrained case, the symmetry significantly decreased, thereby affecting the moiré patterns and conduction and valence band structures, which leads to ferromagnetism, magnetoelectricity, and other properties.

#### 5.1.3 Dielectric Environment

When two graphene flakes are stacked at a small twist angle, the resulting weak and flat dispersing bands strongly enhance electron–electron interactions, which dominate over kinetic energy. Thus, a series of interesting physical properties were generated.^[^ ^88^ ^]^ Beyond the strong effects of the twist angle and strain discussed above, the encapsulated *h*‐BN dielectric may also induce various physical properties, such as unconventional superconductivity and an insulating state,^[^ ^13^, ^151^ ^]^ a quantum anomalous Hall effect, ^[^ ^26^ ^]^ magnetoelectricity,^[^ ^152^ ^]^ and tunable excitons.^[^ ^35^ ^]^ In particular, the alignment^[^ ^153^ ^]^ between tBLG and *h*‐BN and the thickness^[^ ^154^ ^]^ of *h*‐BN play significant roles in regulating these physical properties based on correlated states, which reveal the importance of the microscopic dielectric environment.

**Figure 10:**
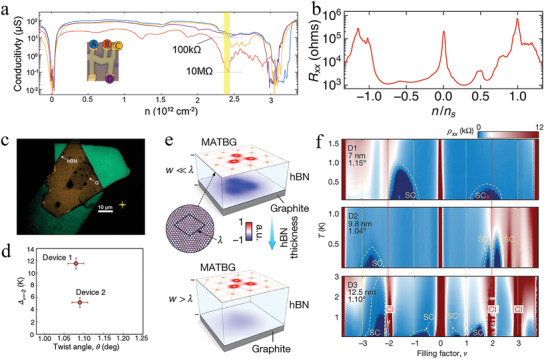
Influence of dielectric environment in the graphene‐based moiré heterostructures. a) Density dependence of the contact conductivity of the four contacts. Reproduced with permission.^[^ ^26^ ^]^ Copyright 2020, American Association for the Advancement of Science. b) Longitudinal resistance *R*_xx_ versus *n* at *D*/*ϵ*_0_ = −0.22 V nm^−1^. c) Optical image of the tBLG heterostructure. b,c) Reproduced with permission.^[^ ^89^ ^]^ Copyright 2019, American Association for the Advancement of Science. d) The comparing of activation gap at *ν* = −2. Reproduced with permission.^[^ ^79^ ^]^ Copyright 2020, Springer Nature. e) Screening‐controlled MATBG phase diagrams for near‐magic twist angles. f) Schematic images of *ρ*_xx_ versus *ν* and *T* for three screening‐controlled MATBG devices. SC refers to superconductivity. e,f) Reproduced with permission.^[^ ^156^ ^]^ Copyright 2020, Springer Nature.

A series of studies on *h*‐BN as a dielectric material were launched to explore the tuning effect of the dielectric environment on the correlated states. For example, the quantum anomalous Hall effect was realized in tBLG aligned to *h*‐BN.^[^ ^26^ ^]^ Consequently, the electrons were polarized into a single spin moiré miniband with Chern number C = 1, and the bandwidth of the Chern bands were tuned to be particularly narrow.^[^ ^24^, ^99^ ^]^ Additionally, a large quantum anomalous Hall effect was observed at a 3/4 filling (highlighted in yellow) of the conduction band with the magic angle tBLG aligned with the top layer of the *h*‐BN substrate (contact B) (**Figure** **10**a).^[^ ^26^ ^]^ The longitudinal resistance of contact B was two orders of magnitude than that of other contacts close to 10 MΩ at 3/4 filling, which was attributed to the break in the in‐plane twofold rotation symmetry and all electrons fill in a Chern band, owing to the alignment of tBLG with *h*‐BN. There was also a significant quantum anomalous Hall effect at ≈10.4 kΩ at 3/4 filling with regards to *R*_xx_‐*n*, as well as another unexpected shoulder peak on the peak of *n* = −*n*_s_ under the condition of *D*/*ϵ*_0_ = −0.22 V nm^−1^ at *n*/*n*_s_ = −1.15 (Figure 10b).^[^ ^89^ ^]^ This peak did not correspond to tBLG alone, but is attributed to the lattice alignment of the top layer graphene and *h*‐BN with an angle of *θ* = 0.81° ± 0.02°.^[^ ^3^ ^]^ Such near‐alignment of tBLG to the top *h*‐BN layer was depicted in an optical image (Figure 10c).^[^ ^89^ ^]^ Collectively, the large quantum anomalous Hall effect ascribed to the C_2_T (where C_2_ refers to a twofold rotation and T is time reversal) breaking that is induced by the alignment between tBLG and *h*‐BN.

In addition to the effect of alignment, electron–electron interactions may also play an indispensable role regarding the physical properties.^[^ ^154^, ^155^, ^156^ ^]^ To investigate the role of electron–electron interactions, superconducting tBLG‐based devices with different *h*‐BN thicknesses were fabricated.^[^ ^79^ ^]^ In these devices, the Coulomb interactions could be screened to a large degree with the varying thickness of *h*‐BN. For example, the superconducting tBLG devices 1 and 2 had an identical twist angle of ≈1.08°; however, the thickness of the *h*‐BN was one order of magnitude different (device 1: *θ* = 1.08 ± 0.02°, *d_h_*_‐BN_ = 68 nm; device 2: *θ* = 1.09 ± 0.02°, *d_h_*_‐BN_ = 6.7 nm). Although both the devices exhibited similar superconducting transition temperatures, device 2 presented a correlated insulating state with an activation gap that was half as wide as that of device 1 (Figure 10d).^[^ ^79^ ^]^ Consistent with this study, Stepanov and co‐workers queried previous research on the coexistent relationship of superconducting and insulating correlated states in the tBLG system^[^ ^92^, ^157^, ^158^ ^]^ and proposed that the superconducting and insulating states in tBLG can be controlled independently by tuning the electronic interactions through changing the distance between the graphene and metal layers.^[^ ^156^ ^]^ Previous studies proved that correlated insulating states appeared under the condition *U*/*t* ≫ 1.^[^ ^12^, ^159^ ^]^ The on‐site Coulomb energy *U* decreased independently as the distance *W* reduced between tBLG and a metallic layer, that is, the thickness of *h*‐BN was reduced. The kinetic energy *t* was increased by tuning the twisted angle *θ* away from the magic angle *θ*_m_ = 1.10°.^[^ ^156^ ^]^ Additionally, the Coulomb interactions were significantly screened by tuning the distance *W* to be smaller than the moiré unit cell size *λ*, *W* < *λ* ≈ 15 nm (Figure 10e). The phase diagrams of the three representative samples of MATBG/*h*‐BN (Figure 10f) with different parameters marked in the diagram show that the correlated insulators (CI) were strongly suppressed for an *h*‐BN thickness *W* < 10 nm, and the deviation of *θ* away from 1.1° was ≈±0.05°. Similarly, by inserting a layer of *h*‐BN between the MATBG and Bernal BLG, the strength of the electron−electron Coulomb interaction in MATBG was continuously adjusted using the charge screening from the Bernal BLG.^[^ ^160^ ^]^ The application of the screening effect weakened the Coulomb interaction, which resulted in a deterioration of the insulating states and an enhanced superconducting stability. These observations indicate that the superconducting and insulating characteristics do not coexist but are in competition. In summary, the thickness of *h*‐BN plays a leading role in tuning electronic interactions to further impact the correlated states.

### 5.2 Correlated Physical Performances

Based on the special moiré superlattice, energy band structures, and the precisely controlled influence factors, graphene‐based moiré heterostructures exhibit fascinating physical properties, such as correlated states, including unconventional superconductivity and insulating states,^[^ ^13^, ^151^ ^]^ ferroelectricity,^[^ ^28^ ^]^ ferromagnetism,^[^ ^34^ ^]^ quantum Hall states,^[^ ^161^ ^]^ the Pomeranchuk effect,^[^ ^27^ ^]^ and tunable excitons.^[^ ^35^ ^]^ Discovering and understanding these phenomena are of great significance to the development of condensed matter physics.

#### 5.2.1 Superconductivity and Insulating States

**Figure 11:**
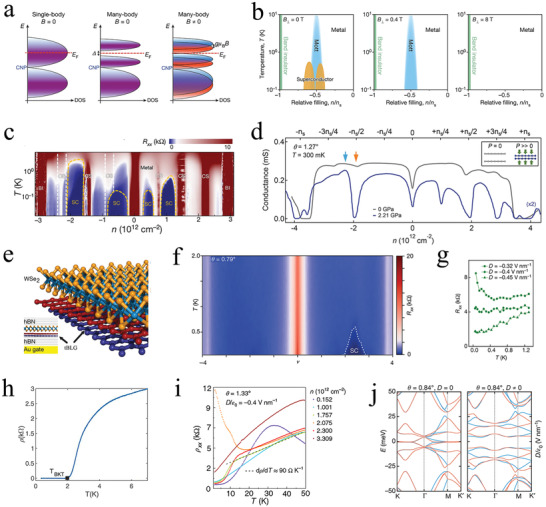
The superconductivity and insulating states in the graphene‐based moiré heterostructures. a) Schematic images of the density of states (DOS) in different scenarios. Reproduced with permission.^[^ ^12^ ^]^ Copyright 2018, Springer Nature. b) Schematic image of the phase diagrams in different magnetic fields. Reproduced with permission.^[^ ^13^ ^]^ Copyright 2018, Springer Nature. c) Resistance as a function of *n* and *T*. Reproduced with permission.^[^ ^21^ ^]^ Copyright 2019, Springer Nature. d) Conductance measured over the entire density range at two values of pressure: 0 GPa (gray) and 2.21 GPa (blue) at *T* = 300 mK. Reproduced with permission.^[^ ^88^ ^]^ Copyright 2019, American Association for the Advancement of Science. e) Schematic image of the tBLG/WSe_2_ heterostructure. f) Longitudinal resistance *R*_xx_ versus *T* and *n*. e,f) Reproduced with permission.^[^ ^165^ ^]^ Copyright 2020, Springer Nature. g) *R*_xx_‐*T* behavior of different electronic states. Reproduced with permission.^[^ ^14^ ^]^ Copyright 2019, Springer Nature. h) Superconducting transition in resistivity. Reproduced with permission.^[^ ^15^ ^]^ Copyright 2021, American Association for the Advancement of Science. i) Longitudinal resistance *ρ*_xx_ versus *T* curves at various *n*. Reproduced with permission.^[^ ^114^ ^]^ Copyright 2020, Springer Nature. j) Schematic images of the calculated energy band structure in the tDBG. Reproduced with permission.^[^ ^10^ ^]^ Copyright 2020, Springer Nature.

Graphene‐based moiré heterostructures, such as MATBG, exhibit strong electron−electron interactions, which result in flat energy bands near the zero Fermi energy and cause a series of correlated phenomena.^[^ ^162^, ^163^, ^164^ ^]^ Correlation refers to the instantaneous interaction between electrons, that is, two or more valence electrons appearing around an atom cause a strong interaction. When electrons form a pair through an attractive interaction, the electron pair can coherently be condensed to form a superconductor. Clearly, correlated insulator states and unconventional superconductivity are the typical correlation states. In the schematic illustrations of the DOS in different cases of correlated insulator states and superconductivity (**Figure** **11**a),^[^ ^12^ ^]^ the single‐particle flat bands with *E*_F_ in the *E* > 0 band was split into many‐body bands by electron–electron interactions, which result in the transformation of the system state from metal to a Mott‐like insulator state at half‐filling and zero magnetic field. Subsequent to applying a Zeeman field (*B* ≠ 0), the excitations of correlated states were polarized, and charge conduction then occurred when the Zeeman energy *gμ*_B_ *B* was equivalent to the charge gap. Inspired by this model, tuning correlated properties has attracted wide interest.

The superconducting phases and correlated insulator phases may be tuned by changing the temperature, magnetic field, and carrier density. For example, the superconductor–insulator–metal transition at different magnetic fields was presented (Figure 11b).^[^ ^13^ ^]^ Notably, there was a transition from metal to superconductor with a decrease in temperature at *B*_⊥_ = 0 T. As the magnetic field increased, the correlated insulator transitioned to a metal phase, owing to the increase in the Zeeman field, and the superconductor transition temperature *T*_c_ reached 1.7 K at the finite twist angle and carrier density.^[^ ^13^ ^]^The transfer process was optimized to reduce the relaxation of the twist angle to realize superconductivity at higher temperatures.^[^ ^21^ ^]^ Although a series of superconducting domes were observed at a similar carrier density, the superconductor transition temperature *T*_c_ was almost 3 K higher than previously reported (*T*_c_ = 1.7 K) (Figure 11c).^[^ ^21^ ^]^ The abovementioned superconducting states were realized near the magic angle, which undoubtedly increased the difficulty of device preparation. Some studies attempted to observe the superconducting states in a larger twist angle range. For example, Yankowitz et al. fabricated a superconducting tBLG‐based device with a twist angle of 1.27°, and they then adjusted the interlayer spacing using hydrostatic pressure to introduce the correlated states.^[^ ^88^ ^]^ From the measurement result of conductance *G* versus carrier density (Figure 11d), strongly insulating states appeared at ±*n*_s_/2 and +3*n*_s_/4 under the hydrostatic pressure *P* = 2.21 GPa. Conversely, no strongly correlated states were observed when *P* = 0 GPa, which indicated that strong correlations were not supported at this twist angle of 1.27°. This difference may have arisen because adjusting the hydrostatic pressure changed the moiré superlattice and energy band structure. This study indicated that graphene‐based moiré heterostructures may be a tunable platform that can be used to explore correlated states by applying larger pressure at larger angles. Starting by decreasing the twist angle, Arora et al. fabricated a series of superconducting tBLG‐based devices with the twist angle lower than the magic angle.^[^ ^165^ ^]^ Given the fact that the insulating layer of *h*‐BN strongly affected correlated states by changing its alignment and thickness, a new moiré heterostructure based on *h*‐BN–tBLG–WSe_2_–*h*‐BN was investigated (Figure 11e).^[^ ^165^ ^]^ A transition metal dichalcogenide, such as WSe_2_, was used as a high‐quality insulating dielectric, similar to *h*‐BN in tBLG‐based devices. The WSe_2_ has many characteristics different from *h*‐BN, such as lattice constants and spin–orbit interaction via vdW and hybridization effects.^[^ ^166^, ^167^, ^168^, ^169^ ^]^ In particular, the monolayer WSe_2_ took advantage of the large bandgap to allow the device a larger range of gate voltages compared with *h*‐BN.^[^ ^170^ ^]^ Moreover, the introduction of WSe_2_ stabilized superconductivity at the smallest twist angle of 0.79° (Figure 11f),^[^ ^165^ ^]^ which is much smaller than the previous observation of superconductivity at 0.93°.^[^ ^139^ ^]^ In addition, superconductivity independently survived at 0.79° without coexisting with an insulating state. This suggests different origins of the two correlated phenomena, which is in agreement with the above introduction.^[^ ^156^ ^]^

Similarly, the superconductivity and correlated insulating states were also discovered in other graphene‐based moiré heterostructures, such as ABC‐TLG,^[^ ^24^ ^]^ magic‐angle trilayer graphene (MATLG),^[^ ^15^ ^]^ and tDBG.^[^ ^115^ ^]^ From the *R*_xx_ versus *T* behaviors in ABC‐TLG (Figure 11g),^[^ ^14^ ^]^ MATLG (Figure 11h),^[^ ^15^ ^]^ and tDBG (Figure 11i),^[^ ^114^ ^]^ the states of a superconductor, insulator, and metal were achieved in these heterostructures via tuning the values of *D* or the carrier density *n*. Moreover, it was not clearly observed that the typical characteristic of the superconductor with increasing temperature is the “freeze” electrons, that is, the resistance increases with the increase in temperature.^[^ ^171^ ^]^ Multiflat‐band systems were different from tBLG, tTLG, and tDBG, which could further flatten the bands and separate them from each other. The band structure of tDBG (Figure 11j) showed that reducing the twist angle to 0.84° resulted in three pairs of flat bands.^[^ ^10^ ^]^ This indicated that all electrons in the range of −3*n*_s_ to +3*n*_s_ may experience strongly correlated interactions, and the interaction strength can be regulated by *D*. The generalization of correlated behaviors from tBLG to tDBG established a tunable platform for exploring quantum many‐body states.

#### 5.2.2 Quantum Anomalous Hall Effect and Ferromagnetism

**Figure 12:**
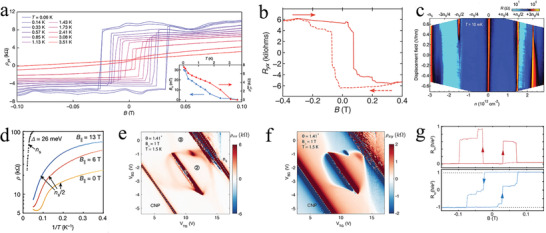
The quantum anomalous Hall effect and ferromagnetic in the graphene‐based moiré heterostructures. a) Schematic image of quantum anomalous Hall effect in the ABC‐TLG system. Reproduced with permission.^[^ ^98^ ^]^ Copyright 2020, Springer Nature. b) Magnetic field dependence of Hall resistance *R*_yx_. Reproduced with permission.^[^ ^89^ ^]^ Copyright 2019, American Association for the Advancement of Science. c) Resistance as a function of displacement field. Reproduced with permission.^[^ ^88^ ^]^ Copyright 2019, American Association for the Advancement of Science. d) Arrhenius plot of the insulating state under different in‐plane magnetic fields. e,f) Longitudinal resistivity (e) and Hall resistance (f) as a function of gate voltage around half‐filling. d‐f) Reproduced with permission.^[^ ^16^ ^]^ Copyright 2020, Springer Nature. g) *R*_xx_ and *R*_xy_ measured at 3/4 filling as a function of magnetic field B. Reproduced with permission.^[^ ^26^ ^]^ Copyright 2020, American Association for the Advancement of Science.

The strong electron−electron interactions in graphene‐based moiré heterostructures actuated not only the superconductivity and correlated states but also the quantum anomalous Hall effect and ferromagnetism under suitable conditions. When comparing the quantum Hall effect with the quantum anomalous Hall effect, it was observed that the quantum Hall effect was generated in 2D electron systems under a strong magnetic field with a finite Chern number *C* and chiral edge states.^[^ ^172^, ^173^ ^]^ Conversely, the presence of the quantum anomalous Hall effect depends on the spontaneous magnetization of the material itself, instead of the external strong magnetic field.^[^ ^174^ ^]^ Moreover, at the finite Chern number *C* = 2, the Hall resistance was well quantized, and it resulted in a ferromagnetic Chern insulator, owing to the strongly enhanced electron−electron interactions, which exhibited a significant magnetic hysteresis and a large anomalous Hall signal at zero magnetic field (**Figure** **12**a).^[^ ^98^ ^]^ This ferromagnetic property, which is induced by electron−electron interactions, can be achieved by adjusting the topological minibands’ structure to lift the spin and valley degeneracy by applying *D*. The quantum anomalous Hall effect was generated when the total nonzero Chen number was achieved, which enabled the investigation of the quantum anomalous Hall effect and ferromagnetic property.

As an intensively studied problem in condensed matter physics, the realization of quantum phenomena was attempted in various graphene‐based moiré heterostructures, such as tBLG,^[^ ^26^, ^175^ ^]^ ABC‐TLG/*h*‐BN,^[^ ^98^ ^]^ and tDBG.^[^ ^16^ ^]^ In these systems, *D* enabled the tuning of the bandwidth and Chern numbers to realize multiple many‐body phenomena, including the quantum Hall effect, quantum anomalous Hall effect, and ferromagnetic property. Taking tBLG as an example, magneto transport was hysteretic in a narrow carrier range at 3/4 filling when an out‐of‐plan magnetic field was applied (Figure 12b). The anomalous Hall resistance reached ±6 kΩ at zero magic field and depended on the sweep direction of the magnetic field, which indicated the existence of remanent magnetization.^[^ ^89^ ^]^ Moreover, a recent study on tBLG has reported high resistance in a narrow range of carrier densities near 3/4 filling (Figure 12c), which suggests the spontaneous breaking of spin and valley symmetries.^[^ ^88^ ^]^ Thus, it is clear that the break in symmetry at 3/4 filling causes a quantized Chern insulator, which results in the quantum anomalous Hall effect and ferromagnetic property.

As the number of layers increases, the conditions required to realize the quantum phenomena change, owing to the varying of the moiré superlattice and energy band structure, such as the twist angle, carrier densities, and *D*. In tDBG, the energy gaps at the half filling increased with the in‐plane magnetic field (Figure 12d), which indicated a ferromagnetic order, owing to the gaps; this indicated the emergence of correlated insulator states.^[^ ^16^ ^]^ Correspondingly, the measured Hall effect presented as a typical quantum Hall effect. There was a clear change in sign at 1/2 filling, which indicated that the longitudinal resistance reached zero within the carrier density (Figure 12e). Meanwhile, the Hall resistance was maintained at a fixed value (Figure 12f). The appearance of the quantum Hall platform under the condition of zero longitudinal resistance suggested the realization of the quantum Hall effect, which is caused by spin polarization and results in a smaller energy bandgap. The discovery of ferromagnetism and the quantum Hall effect in electric‐field‐tunable tDBG enabled engineering interaction‐driven quantum phases.

Hall phenomena were observed not only in the twisted graphene, but also in the twisted graphene/*h*‐BN heterostructure. From the magnetic field dependence of *R*_xx_ and *R*_xy_ at 3/4 filling in tBLG/*h*‐BN heterostructures (Figure 12g), the *R*_xy_ was quantized to *h*/*e* ^2^ concomitant with a minimum *R*_xx_.^[^ ^26^ ^]^ Typically, *R*_xy_ was hysteretic and the coercive field reached several tens of millitesla. Similar to twisted graphene, the effect was driven by spontaneously breaking the time‐reversal symmetry. The discovery of the quantum anomalous Hall effect and ferromagnetic property promoted the development of electronic devices utilized in daily life because the quantum anomalous Hall effect could be realized under zero magnetic field.

#### 5.2.3 Ferroelectricity and Magnetoelectricity

**Figure 13:**
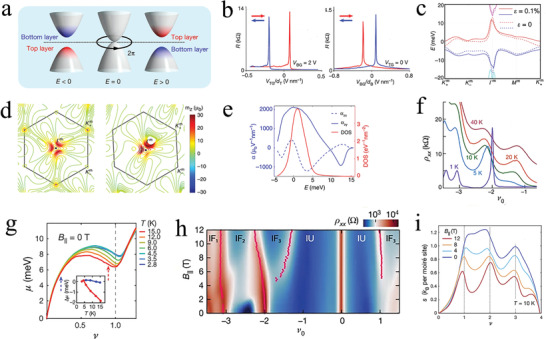
The ferroelectricity, magnetoelectricity, and Pomeranchuk effects in the graphene‐based moiré heterostructures. a) Schematic images of the band dispersion and layer polarization of the BLG at different interlayer electric fields. b) Four‐probe resistance in the BLG showing substantial hysteretic. a,b) Reproduced with permission.^[^ ^28^ ^]^ Copyright 2020, Springer Nature. c) The flat bands energy dispersion from the *K*_+_ valley with the twist angle *θ* = 1.2° with strain *ε* = 0 and *ε* = 0.1%. d) The orbital magnetic moments of the Bloch electrons with strain *ε* = 0 and *ε* = 0.1%. e) The magnetoelectric response in strained TBG. c‐e) Reproduced under the terms of the Creative Commons CC‐BY license.^[^ ^177^ ^]^ Copyright 2020, The Authors. Published by Springer Nature. f) The *ρ*_xx_ as a function of temperature *T* and *ν*_0_. Reproduced with permission.^[^ ^178^ ^]^ Copyright 2021, Springer Nature. g) The chemical potential *μ* as a function of temperature *T* and *ν*_0_. Reproduced with permission.^[^ ^27^ ^]^ Copyright 2021, Springer Nature. h) The *ρ*_xx_ as a function of *ν*_0_ and *B*_∥_ at *T* = 20 mK. Reproduced with permission.^[^ ^178^ ^]^ Copyright 2021, Springer Nature. i) The electronic entropy *s* as a function of *ν* and *B*_∥_ at *T* = 10 K. Reproduced with permission.^[^ ^27^ ^]^ Copyright 2021, Springer Nature.

In graphene‐based moiré heterostructures, the strong electron−electron interactions spontaneously break symmetry, which leads to various properties. In detail, the spontaneous breaking of symmetry including gauge symmetry, spin rotational symmetry, and point group symmetry, plays an important role in the study of novel phases in condensed‐matter physics. For example, the spontaneous breaking of gauge symmetry introduced superconductivity,^[^ ^88^ ^]^ and the spontaneous breaking of spin rotational symmetry resulted in magnetism.^[^ ^98^ ^]^ However, the effect of point group symmetry in graphene‐based moiré heterostructures can introduce ferroelectricity, but it remains underexplored. A moiré system of Bernal‐stacked BLG aligned with *h*‐BN was investigated to understand the special property of ferroelectricity, owing to its the tunable electronic phases and symmetry according to an external *D*.^[^ ^28^ ^]^ As a typical point group symmetry, space inversion symmetry was introduced, which was equivalent to layer degeneracy. From the illustration of the energy band structure, layer degeneracy was broken, which resulted in a layer polarization. The direction of polarization relied on *D*, which implies the existence of ferroelectricity (**Figure** **13**a).^[^ ^28^ ^]^ In addition, substantial hysteresis between the forward and backward gate voltage scans was observed (Figure 13b). In this case, the *x*‐axis represents the change in *D* because $D_{b}=+\varepsilon_{b}\left(V_{b}-V_{b}^{0}\right)/d_{t}$ and $D_{t}=-\varepsilon_{t}\left(V_{t}-V_{t}^{0}\right)/d_{t}$. This ferroelectric system with strong hysteresis can be applied to high‐performance memory devices.

The ferroelectricity generated by symmetry breaking is unstable; thus, introducing it into magnetic or superconducting systems may cause magnetoelectric coupling.^[^ ^176^ ^]^ Different from the ferromagnetism induced by electron−electron interactions, the magnetoelectric effect results from the charge current at general filling factors; even the tBLG sample is not ferromagnetic. In the tBLG system, the symmetry reduced to *D*_6_ (chiral point group) because twisting allowed the charge current to induce magnetization.^[^ ^152^ ^]^ However, it is difficult to generate out‐of‐plane magnetization via an in‐plane current. Thus, a tBLG/*h*‐BN model was induced to decrease the symmetry, which aimed to generate out‐of‐plane magnetization under a small in‐plane current. In this case, the *h*‐BN substrate had two functions: 1) it broke the *C*_2_ symmetry, resulting in a massive gap (Figure 13c); 2) it exerted strain on the bottom graphene layer, leading to a crystal symmetry decrease in *C*_1_ (Figure 13d).^[^ ^177^ ^]^ Consequently, the magnetoelectric susceptibilities were tuned by the Fermi energy, which caused a magnetoelectric response in strained tBLG (Figure 13e). In summary, the appearance of the magnetoelectric effect is primarily due to the breaking of symmetry and the application of strain. Therefore, in other graphene‐based moiré heterostructures with low crystal symmetries, such as the tDBG,^[^ ^179^ ^]^ graphene/*h*‐BN,^[^ ^180^ ^]^ and graphene/TMDCs,^[^ ^124^ ^]^ similar magnetoelectric performance may be induced by applying strain.

#### 5.2.4 Pomeranchuk Effect

The strong electron−electron interactions produce correlated insulating states, which stems from a change in the electron states in the graphene‐based moiré heterostructures. In this moiré flat‐band system, the electron was frozen from a Fermi liquid (metal) to a solid (insulator) as the temperature increased, which resulted in the Pomeranchuk effect.^[^ ^12^, ^13^, ^32^ ^]^ The Pomeranchuk effect was first observed in ^3^He, in which a high excess nuclear spin entropy‐driven transition occurs between a liquid at low temperature and a solid at high temperature.^[^ ^181^ ^]^ Unexpectedly, an analogous effect appeared in the MATBG.^[^ ^12^, ^13^, ^32^ ^]^ In the MATBG consisting of two stacked but slightly misaligned graphene sheets, the isospin‐unpolarized Fermi liquid (metal) was frozen to a solid (insulator) as the temperature increased. The electrons underwent a process from disordered movement in the metal phase to a near‐insulating phase, in which the electron position was zero. Moreover, the near‐insulating phase has higher entropy (disorder) than metal; therefore, the electrons were frozen at a high temperature.^[^ ^171^ ^]^ The Pomeranchuk effect was adjusted by tuning the ratio between the electronic interaction and bandwidth. The Mott insulators were formed because the electrons tended to localize when the electronic interaction was dominant. However, a Fermi liquid state was formed. At a finite electronic filling of the flat bands at *ν* ≈ −1 (Figure 13f)^[^ ^178^ ^]^ and at *ν* ≈ 1 (Figure 13g),^[^ ^27^ ^]^ the MATBG states converted metal to a near‐insulating phase with high‐resistance, which indicated the presence of the Pomeranchuk effect. The connection between the high‐temperature phase and the breaking of isospin‐symmetry was illustrated to explore the origin of this special phenomenon (Figure 13h).^[^ ^178^ ^]^ The parallel magnetic field *B*_∥_ was observed to partially polarize the isospin electron to form a large magnetic moment. This valley‐polarized isospin ferromagnetic (IF_3_) state at high *B*_∥_ is in good agreement with the illustration of the above‐mentioned Pomeranchuk effect. Additionally, the entropy of the near‐insulating phase also strongly depends on *B*_∥_, in which most of the entropy was quenched at *ν* ≈ 1 (Figure 13i).^[^ ^27^ ^]^ Overall, the appearance of the IF_3_ state and the decrease in entropy in the near‐insulating phase reasonably explains the origin of the Pomeranchuk effect. However, this new finding also leaves many questions, such as the mechanism of the phase transition and the absence of the Pomeranchuk effect in other electronic fillings. It is believed that additional novel physical properties of graphene‐based moiré heterostructures will be discovered as these problems are solved.

#### 5.2.5 Excitons

**Figure 14:**
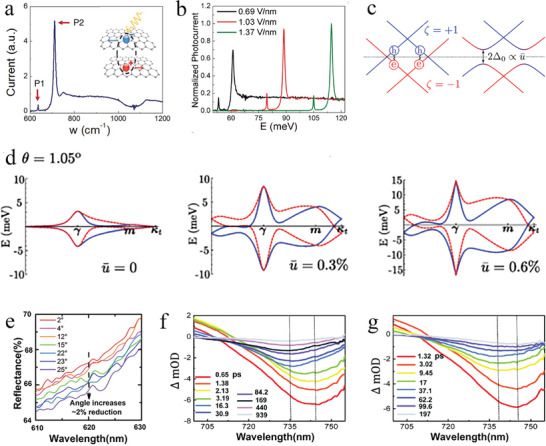
The excitons states in the graphene‐based moiré heterostructures. a) Photocurrent spectrum of the BLG. The inset illustrates the photocurrent generation process. b) Normalized photocurrent spectrum at different displacement fields. a,b) Reproduced with permission.^[^ ^35^ ^]^ Copyright 2017, American Association for the Advancement of Science. c) Schematic image of shifting the Dirac points in energy via applying a strain. d) Schematic images of the lowest‐energy bands for magic angle at different strain. c,d) Reproduced with permission.^[^ ^185^ ^]^ Copyright 2020, American Physical Society. e) Reflectance spectra of the graphene/MoS_2_ graphene heterostructure with different twist angles. Reproduced with permission.^[^ ^29^ ^]^ Copyright 2019, AIP Publishing. f) The transient absorption spectra of the WSe_2_/graphene heterostructure. g) The transient absorption spectra of the graphene/WSe_2_ heterostructure. f,g) Reproduced with permission.^[^ ^186^ ^]^ Copyright 2020, The Royal Society of Chemistry.

In addition to the generation of the Pomeranchuk effect, the changing of electronic behaviors also produces excitons. Excitons are the bound states of an electron and a hole in semiconductors, which are generated by exciting an electron from the full valence band to the empty conduction band to form a tightly bound electron−hole pair. The exciton effect has an important influence on physical processes, such as photoluminescence emission,^[^ ^182^ ^]^ light absorption,^[^ ^183^ ^]^ and optical nonlinear effects^[^ ^184^ ^]^ in semiconductors; thus, the exciton effect plays a vital role in optical properties. Despite practical interest, it is difficult to observe excitons. Graphene‐based moiré heterostructures are a class of materials with a continuously tunable bandgap and pseudospin texture, which provide a platform for the observation of excitons.^[^ ^114^ ^]^ For example, electron−hole pairs were generated in the *h*‐BN‐encapsulated BLG moiré heterostructures under incident light, which resulted in a photocurrent with two absorption peaks that were proportional to the optical absorption (**Figure** **14**a).^[^ ^35^ ^]^ These photocurrent peaks shifted to higher energies as the bandgap increased, owing to the application of *D* (Figure 14b), which reflected the existence of a tunable photocurrent.^[^ ^35^ ^]^ In addition to the regulation of the exciton state by *D*, the layer‐asymmetric strain also had a significant influence on this effect because the strain assisted the formation of the energy gap. When strain was evident in tBLG, the electron−hole symmetry was broken and the Dirac points shifted in energy (Figure 14c), which indicated the effect of strain on the excitonic gap.^[^ ^185^ ^]^ In the MATBG system, the bandgap between the valence band and the conduction band widened with increasing strain (Figure 14d), which further proved the influence of strain on the exciton effect.^[^ ^185^ ^]^ Moreover, the exciton effect also appeared in the graphene/TMDCs, such as graphene/MoS_2_, which showed that the exciton absorption is dependent on the twist angle.^[^ ^29^ ^]^ As the twist angle increased, the interlayer coupling declined. Correspondingly, the exciton formation process was promoted to facilitate exciton absorption, owing to the decreasing interlayer electron transfer (Figure 14e), where a wavelength of 620 nm corresponded to the photoluminescence peak of MoS_2_.^[^ ^29^ ^]^ Additionally, the exciton lifetimes also exhibited a significant difference when the stacking order of graphene and TMDCs was varied. From transient absorption spectroscopy, the exciton lifetime of the WSe_2_/graphene

heterostructure (Figure 14f) was considerably higher than that of the graphene/WSe_2_ heterostructure (Figure 14g), owing to the difference in charge screening of the substrate.^[^ ^186^ ^]^ To summarize, the exciton effect has been observed through the variation of the photocurrent in graphene‐based moiré heterostructures, which has a significant influence on the optical properties. The exciton formation process and lifetime rely on the structure of graphene‐based moiré heterostructures, such as the twist angle and the stacking order of different layers. These results enrich the understanding of the exciton effect in graphene‐based moiré heterostructures, which provides significant guidance toward designing optoelectronic devices.

## 6 Summary and Outlook

In the field of condensed matter physics, graphene‐based moiré heterostructures have been extensively studied as a promising platform to implement the regulation of moiré pattern and energy band structures and the observation of correlated phenomena. In particular, the realization of a superflat miniband and the transition between correlated insulator states and superconductivity promotes the development of twistronics.

Herein, we have presented the discovery and development of various topological performances and correlated physical properties in graphene‐based moiré heterostructures. The foundation of this system is the effective preparation of graphene‐based moiré heterostructures. We have briefly described fabrication methods, including in situ growth and the assembling of monolayer 2D materials. Then, we summarized the methods that may be used to enhance the quality of graphene‐based moiré heterostructures, including the optimization of the transfer process and improvement of graphene quality. Finally, the introduction of properties and influence factors enable further research on condensed matter physics.

Despite the significant progress reviewed herein, it should be noted that some crucial challenges limit the progress of graphene‐based moiré heterostructures. For example, it is difficult to achieve high‐quality graphene‐based moiré heterostructures via in situ growth or assembly. The control of the twist angle is not precise in in situ growth, and the promising method of the assembling process causes serious tearing, wrinkles, and/or interface polymer residues, which directly limits in‐depth research on the physical properties of these heterostructures and their effective applications to optoelectronic devices. There are additional problems regarding the preparation methods. First, the preparation of tBLG with a specific twist angle may be achieved using CVD; however, there are challenges in directly achieving graphene‐based moiré heterostructures composed of different 2D materials, such as graphene/*h*‐BN and graphene/MoS_2_. Second, it is difficult to fabricate high‐quality graphene‐based moiré heterostructures on a large scale, owing to the difficulty in controlling the twist angle. Moreover, the assembly method inevitably affects the performance of graphene‐based moiré heterostructures, owing to the transfer process and mechanical regulation of the twist angle, which results in a low yield of the heterostructures. Recent advances in the fabrication of graphene‐based moiré heterostructures enable high controllability over the stacking order. It is believed that full control of the twist angle may be achieved in the near future.

In addition to fabrication, the characterization of graphene‐based moiré heterostructures is also a challenge that affects its potential applications.^[^ ^187^ ^]^ Graphene‐based moiré heterostructures must undergo extensive characterization before device application and physical performance exploration, such as TEM,^[^ ^134^, ^188^ ^]^ STM,^[^ ^157^ ^]^ and AFM.^[^ ^189^ ^]^ The direct characterizing of the moiré pattern using TEM requires the samples to be placed on a dedicated TEM grid, which is destructive to the sample. STM may use the probe tip to produce an image of the moiré superlattice; however, the characterization requires the sample to be conductive, which prevents it being packaged by *h*‐BN. Similar to STM, the AFM tip is in contact with the graphene‐based moiré heterostructures, and it requires no top packaging or top gates, which limits device applications. Therefore, it is essential to improve the characterization methods and particularly develop the in situ characterization of moiré superlattices in graphene‐based moiré heterostructures, which may be of great significance.

The opportunities and challenges are always in coexistence. The challenges introduced above also provide opportunities for the advanced research of graphene‐based moiré heterostructures. As the variety of 2D materials increases, an increasing number of moiré heterostructure compositions may be attempted, including, but not limited to, graphene‐based moiré heterostructures. A combination of other 2D materials apart from graphene may be utilized. In addition, the discovery of new moiré heterostructures is likely, which will have profound significance for the design of multi‐function devices and research on physical properties. For example, the supermoiré heterostructure *h*‐BN/graphene/*h*‐BN with slight misalignment between its layers behaves similarly to two interfering moiré patterns, which generates a lattice and electronic spectrum reconstruction.^[^ ^190^ ^]^ Additionally, graphene encapsulated by *h*‐BN can cause electrons to scatter, which leads to spectral changes at arbitrarily low energies; this is beneficial for controlling the properties of 2D materials.^[^ ^191^ ^]^ Stacking two layers of TMDCs with a specific angle to engineer flat bands may realize insulating and superconducting states,^[^ ^192^ ^]^ which provides a platform to exploring other electronic phases such as exciton condensates and spin liquids. The moiré heterostructures composed of *h*‐BN and TMDCs may be applied to investigate correlated electronic states and may also be used as spectroscopic tools to study interaction‐induced exciton‐polarons.^[^ ^6^, ^193^ ^]^ Graphene encapsulated by hexagonal *h*‐BN and slightly misaligned with the top and bottom *h*‐BN layers experiences two interfering moiré patterns, which results in a so‐called supermoiré. Recently, extensive research has been conducted on graphene‐based moiré heterostructures to understanding many‐body physics; however, the mechanism of the superconducting phase remains unclear.^[^ ^194^ ^]^ It is believed that the investigation of graphene‐based moiré heterostructures will provide rich research opportunities in the near future.

## Conflict of Interest

The authors declare no conflict of interest.
